# Mechanistic study of attenuation of monosodium glutamate mixed high lipid diet induced systemic damage in rats by *Coccinia grandis*

**DOI:** 10.1038/s41598-020-72076-6

**Published:** 2020-09-22

**Authors:** Arnab Banerjee, Debasmita Das, Rajarshi Paul, Sandipan Roy, Ujjal Das, Samrat Saha, Sanjit Dey, Arghya Adhikary, Sandip Mukherjee, Bithin Kumar Maji

**Affiliations:** 1 Department of Physiology (UG & PG), Serampore College, 9 William Carey Road, Serampore, Hooghly-712201, West Bengal India; 2 Department of Physiology, University College of Science, Technology and Agriculture, University of Calcutta, 92 Acharya Prafulla Chandra Road, Kolkata-700009, West Bengal India; 3 Centre for Research in Nanoscience and Nanotechnology, Acharya Prafulla Chandra Roy Sikhsha Prangan, University of Calcutta, JD-2, Sector-III, Saltlake City, Kolkata-700098, West Bengal India

**Keywords:** Physiology, Health care

## Abstract

In the context of failure of treatment for non alcoholic fatty liver disease (NAFLD)-mediated systemic damages, recognition of novel and successful characteristic drug to combat these anomalous situations is earnestly required. The present study is aimed to evaluate protective value of ethanol extract of *Coccinia grandis* leaves (EECGL), naturally occurring medicinal plant, on NAFLD-mediated systemic damage induced by high lipid diet along with monosodium glutamate (HM)-fed rats. Our study uncovered that EECGL significantly ameliorates HM-induced hyperlipidemia, increased lipogenesis and metabolic disturbances (via up regulation of PPAR-α and PPAR-γ), oxidative stress (via reducing the generation of reactive oxygen species and regulating the redox-homeostasis) and inflammatory response (via regulating the pro-inflammatory and anti-inflammatory factors with concomitant down regulation of NF-kB, iNOS, TNF-α and up regulation of eNOS). Furthermore, EECGL significantly inhibited HM-induced increased population of cells in sub G0/G1 phase, decreased Bcl2 expression and thereby loss of mitochondrial membrane potential with over expression of Bax, p53, p21, activation of caspase 3 and 9 indicated the apoptosis and suppression of cell survival. It is perhaps the first comprehensive study with a mechanistic approach which provides a strong unique strategy for the management of HM-induced systemic damage with effective dose of EECGL.

## Introduction

Nowadays, younger generation children are too much fond of fast food from nearby cafe or from the local restaurant which are exceptionally alluring and extremely fiery too. This food contains saturated fat, hydrogenated fats which are fundamentally found in high lipid diet (HLD)^1^ and in combination with flavor upgrading substance monosodium glutamate (MSG) popularly known as ajinomoto is the silent killer of various organs of animal models and in human. Lack of physical activity or absence of dynamic work with high consumption of the above combinations of food constituents may at last generate situation of dyslipidemia as well as hyperglycemia. These combinations may help in the generation of reactive oxygen species (ROS) and likewise cause asymptomatic non alcoholic fatty liver disease (NAFLD) which ultimately leads to the progression of necrosis, hepatic steatosis, fibrosis, cirrhosis, programmed cell death and finally progression of liver malignancy. In the present time, NAFLD immensely set apart as a basic medical issue everywhere throughout the World which at last prompts cardiovascular disorders (CVD) and non-insulin-dependent diabetes mellitus (NIDDM)^2–4^. Improvement of NAFLD is a convoluted procedure and relying upon a few variables and particularly relating to a condition or ailment coming about because of the cooperation of numerous qualities which depends on the "two-hit" theory and it expresses that either triglycerides or fatty acids aggregated in hepatic tissue, event of insulin resistance (IR), inflammatory response and generation of ROS may cause the hepatic injury. Moreover, recently "multiple-hit model" have been widely acceptable hypothesis instead of the outdated "two-hit" hypothesis; it is mainly based on the relationship between environmental and genetic factors to promote metabolic malfunctions and systemic damage^5^. Actuation of provocative variables may quietly take a part in high lipid diet incited non-alcoholic steatohepatitis (NASH)^1^. Superfluity of reactive oxygen species and other free radicals by various physiological and biochemical processes is generally alluded to oxidative stress (OS) mediated systemic damage. HLD and MSG both are linked with generation of ROS and thereby oxidative stress^1,6^. The doses and administration of MSG in rodents’ model in different investigations intended for obesity, digestive problem, hepatic and cardiac ill effects. These influencing factors are more like human MSG admission than in studies concentrated on MSG related consequences for the central nervous system (CNS)^7^. MSG caused histological alteration like tissue tightening in kidney of rodent model and also actuated damage in liver and kidney^8^. High dietary intake of MSG as flavor enhancer in rat stimulates liver toxicity by alteration of oxidative stress parameters associated with DNA fragmentation and proliferating cell nuclear antigen alterations^9^. Moreover, MSG in high fat diet is reported to promote kidney damage by altering the gut microbiota and this can leads to one of the noncommunicable disorder like chronic kidney disease^10^.

Inappropriate viable treatment in the present day medication, endeavors are being made to discover reasonable home-grown medications. *Coccinia grandis* (L) voigt, commonly known as Ivy gourd with cucurbitaceae is widely available in India, Sri Lanka and Pakistan. All parts of the plant utilized in conventional treatment procedure and it is utilized for liver illnesses, diabetes mellitus, antimicrobial, asthma, ulcer, urinary tract maladies, sensitivity and bronchitis^11^. Nourishment items containing plant compounds are broadly utilized as remedial dietary choices for lessening hypercholesterolemia and the atherosclerotic risk^12–14^. Antioxidants like flavonoids, triterpens and tannin are present in *Coccinia grandis* and may intervene with free radical development affirmed that hepatoprotective activities of specific flavonoids are known^15^. Deshpande et al.^16^ assessed the *Coccinia grandis* leaves and stem have anti-inflammatory activity in rat model. The anticancer activity of *Coccinia grandis* is because of its antioxidant content. Polyphenol in *Coccinia grandis* leaves brings down the plasma lipid profile, expanding high density lipid cholesterol and total cholesterol ratio^17^. Considering their antioxidant, cholesterol lowering, hypoglycemic, pain relieving, antimicrobial, antipyretic, calming, antiulcer, antidiabetic, hepatoprotective, antimalarial, antidyslipidemic, anticancer, antitussive, mutagenic activities^18^ with different bioactive phytoconstituents in particular alkaloid, cardenolides, flavonoids, terpenoids, saponins and polyprenols^19^ are most prominent in *Coccinia grandis* leaves, we estimated that it would weaken the occasions prompting NAFLD mediated hepatic steatosis which may increase the risk of cardiovascular ill effects. To demonstrate this speculation, the present investigation was intended to assess the preventive impacts of ethanol extract of *Coccinia grandis* leaves (EECGL) on NAFLD utilizing animal model nourished in combination of high lipid diet along with monosodium glutamate (HM). Three different concentrations of EECGLs were utilized, which was fed by the experimental animals orally during the experimental period of study. The lipid profile, hepatic and cardiac marker enzymes, hepatic and cardiac lipid content and histopathological changes were observed. Furthermore, the impacts of EECGL on inflammatory factors, oxidative markers were investigated, the genes identified with inflammation and proteins identified with apoptosis were evaluated to further clarify the impacts of EECGL on NAFLD intervened systemic damage.

## Materials and methods

### Chemicals and reagents

Monosodium glutamate (SRL, India), 5,59-dithio-bis (2-nitro benzoic acid) (DTNB), Triton X-100, Trichloroacetic acid (TCA), 4-(2-hydroxyethyl)-1-piperazineethanesulfonic acid (HEPES), Thiobarbituric acid (TBA), Tween 20 and Bovine serum albumin (BSA) were purchased from Sigma Aldrich (USA). Antibodies such as Bcl2, Bax, p21, p53, NF-kB (p65), cleaved caspase 3, cleaved caspase 9 and GAPDH, 4′,6-diamidino-2-phenylindole (DAPI) were purchased from Cell Signalling Technology (USA) and PPAR-α from GeneTex (USA), PPAR-γ from Abcam (USA). Ethidium bromide (EtBr), ethanol, hydrogen peroxide (H_2_O_2_), petroleum ether, chloroform, n-hexane, acetone and all other chemicals were procured from Merck (Germany). Anti-rabbit IgG fluorescein isothiocyanate (FITC) and Radioimmunoprecipitation assay buffer (RIPA) buffer were procured from Santa Cruz Biotechnology (USA). 3-3′-dihexyloxacarbocyanine iodide (DiOC6, Molecular Probes), 2′,7′-dichlorofluorescein diacetate (DCFH-DA) and RNase-PI were procured from Thermo Fisher Scientific (USA). All the cell culture media, buffer, collagenase type II, Trizol reagent and other reagents were obtained from Gibco (USA) and all other reagents used for this study were of highest quality grade.

### Preparation of ethanol extract of *Coccinia grandis* leaves (EECGL)

Fresh mature leaves of *Coccinia grandis* were collected from Serampore College campus and authenticated (Specimen No. SC/HPY/BM/AB/001) by the Botanical Survey of India, Central National Herbarium, Howrah, 711 103, India. The leaves were at first washed with distilled water and shed dried for 15 days, at that point ground into fine powder by electrical processor and sieved through the 40 micron strainer and put away in water or air proof compartments (put away at 4 °C until further use). 100 g of dried and powdered of *Coccinia grandis* leaves was extracted in 500 ml of 99.9% ethanol for 72 h in soxhlet apparatus, and the extract was centrifuged for 15 min at 4,000 rotation per minute (rpm). Supernatant was taken as ethanol extract of *Coccinia grandis* leaves (EECGL), concentrated by using rotary evaporator at 40 °C and dried in lyophilizer. Suitable grouping of concentrate was then diluted with distilled water before being supplemented to the experimental animals. The concentrate was kept at −20 °C for further research work^19^.

### High performance thin layer chromatography (HPTLC)

A high performance thin layer chromatography (Linomat 5) of EECGL was done for identification as well as quantification of the bioactive compound present in it. The solvent was prepared by petroleum ether, n-hexane and acetone as mobile phase in a ratio of 2:3:1 utilizing TLC silica gel plate as adsorbent. After that the samples were measured in the LAMAG TLC scanner 3. The densitometric scanning was performed on Camag TLC Scanner 3 at absorbance 280 nm (D2 light) worked by multi level win CATS planar chromatography director^20^.

### Gas chromatography (GC)/mass spectrometry (MS)

Polaris Q Mass spectrometer combined with Trace GC ultragas chromatography (Thermo Fisher Scientific India Pvt. Ltd.) and Perkin-Elmer Gas Chromatography–Mass Spectrometry were used for more confirmation about the proof of the various compound(s) present in EECGL. The component(s) of EECGL were identified by utilizing Xcalibur^21^.

### Acute toxicity study

Acute oral toxicity study of EECGL was performed according to the guideline 2001 of Organization for Economic Co-operation and Development^22^. For this purpose, overnight fasted 48 male albino rats of Wistar strain were taken and they were divided into six groups (one control group and five treated groups) randomly with 8 rats in each group. Rats were administrated EECGL by oral gavages as increasing doses of 1,000, 2000, 3,000, 4,000 to 5,000 mg/kg body weight. We observed behavioral changes of rats such as restlessness, drowsiness, piloerection, writhing, convulsion and mortality if any for 8 h at an interval of one hour and then for the next 48 h at an interval of 4 h.

### Method of preparation of high lipid diet (HLD)

Edible coconut oil and vanaspati ghee were procured from the local market. Two parts of edible coconut oil and three parts of vanaspati ghee were mixed to prepare HLD (40% of edible coconut oil and 60% of vanaspati ghee as the source of saturated and hydrogenated fats). Banerjee et al*.*^1^ has been well documented that Indians are used coconut oil and vanasppati ghee as cooking oil that contains large quantity of hydrogenated fats and saturated fatty acids such as palmitic acid, lauric acid, myristic acid; major components of coconut oil are saturated fatty acids like lauric acid, myristic acid, caprylic acid, palmitic acid etc. In addition, saturated fatty acid content of vaspati ghee and coconut oil are approximately 44.98% and 30.83% respectively. Further, vanaspati ghee contains large amount of trans fatty acids as compared to coconut oil^1^.

### Method of preparation of monosodium glutamate (MSG)

2.4 g of MSG was dissolved in 40 ml of distilled water in every day just before the treatment period^6^.

### Experimental animals and planning of treatment

**Figure 1:**
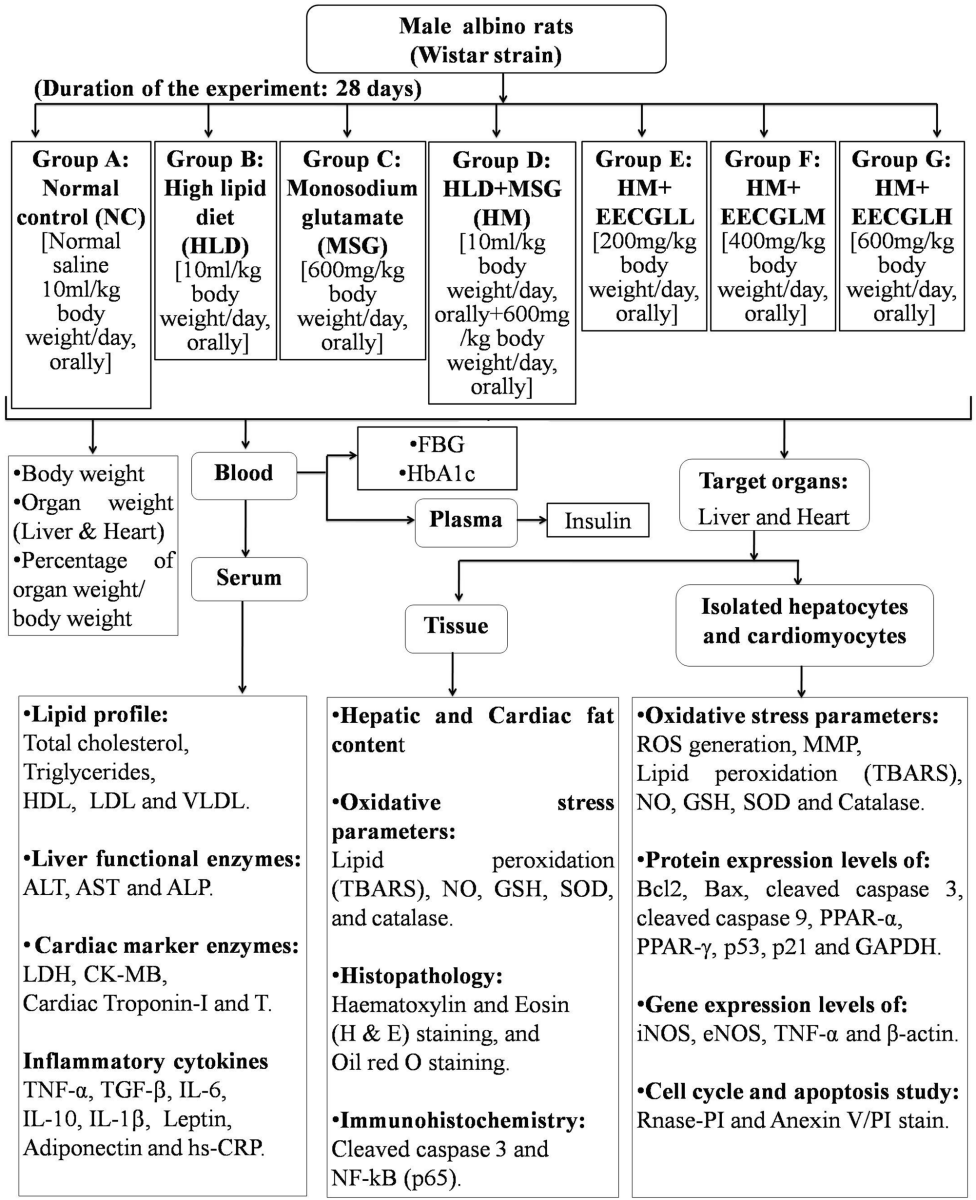
Experimental design. The plan of the experiment was represented by the schematic diagram. Experimental period of the force feeding of HLD, MSG, HM were 28 days with supplementation of EECGLs and a control group (NC). The different experimental parameters were measured after the completion of the experimental period (on or after 29th day).

The experimental design has been given in Fig. 1. All experimental protocols were approved and performed according to the ethical guidelines suggested by Serampore College Institutional Animal Ethics Committee, West Bengal, India (Project Approval No. 07/P/S/IAEC/2017 and 24/P/S/SC/IAEC/2019) registered under Committee for the Purpose of Control And Supervision of Experiments on Animals (CPCSEA), Government of India (Reg. No. 1946/PO/Re/S/17/CPCSEA). No human subject was used in the study. Male albino rats of Wistar strain (110 ± 10 g) were used for the experiment. The duration of the experiment was 28 days. For the experiment, 56 healthy male adult rats were randomly selected and were divided into seven equal groups (n = 8) and treated as: NC as normal control group (normal saline 10 ml/kg body weight/day, orally), high lipid diet group (HLD, 10 ml/kg body weight/day, orally which equates to 600 ml/60 kg of humans respectively), monosodium glutamate group (MSG, 600 mg/kg body weight/day, orally which equates to 3.6 g/60 kg of humans respectively), HLD + MSG group (HM, 10 ml/kg body weight/day, orally + 600 mg/kg body weight/day, orally), HM + low dose of EECGL (EECGLL, 200 mg/kg body weight/day, orally which equates to 1.2 g/60 kg of humans respectively), HM + medium dose of EECGL (EECGLM, 400 mg/kg body weight/day, orally which equates to 2.4 g/60 kg of humans respectively), HM + high dose of EECGL (EECGLH, 600 mg/kg body weight/day, orally which equates to 3.6 g/60 kg of humans respectively). The rats of all groups were supplied with a control diet prepared with 71% carbohydrate, 18% protein, 7% fat, and 4% salt mixture and water ad libitum^1^.

### Body weight, organ weight, percentage of organ weight/body weight, blood collection, serum preparation

24 h of fasting at the end of last administration (on the 29th day) animals were anesthetized with intraperitoneal (ip) ketamine (87 mg/kg body weight) suggested by IAEC. Body weight, organ weight and percentage of organ weight/body weight were calculated. Blood was drawn by cardiac puncture for measuring of blood sugar; plasma and serum was isolated for the different biochemical parameters.

### Fasting blood glucose (FBG)

Bayer Contour TS glucometer (United Kingdom) was utilized to estimate FBG according to the manufacturer’s instructions after the experimental period and on the day of sacrifice of the animal.

### Determination of HbA1c

Ion exchange resin-based method was utilized to estimate glycated haemoglobin (HbA1c) by using spectrophotometer.

### Determination of insulin

Enzyme linked immunosorbent assay (ELISA) was utilized to measure plasma insulin of rat by using standard kit from Cayman chemicals (USA) and was expressed in μIU/ml.

### Serum biochemical parameters

Serum biochemical parameters were estimated by earlier published method of Banerjee A et al. (2020) with some basic modifications^1^. Aspartate transaminase (AST), alkaline phosphatase (ALP), alanine amino transaminase (ALT), total cholesterol (TC), triglycerides (TG), low density lipoprotein (LDL), very low density lipoprotein (VLDL), and high density lipoprotein (HDL) were estimated by standard kit from Accurex Biomedical Pvt. Ltd. (Mumbai, India). Automated immunoturbidimetric assay was performed to measure high sensitivity C-reactive protein (hs-CRP) level (Cobas Integra 800, Roche diagnostics, Switzerland) by enzyme immunoassay.

Serum lactate dehydrogenase (LDH) level was measured by using standard kit from Stanbio Laboratory (USA) and reading was estimated by PD-303S spectrophotometer (APEL, Japan); enzyme-linked immunosorbent assay kit (Thermo Fisher Scientific, Waltham, MA, USA) was used to estimate creatine kinase-MB (CK-MB) as per the instruction of the manufacturer. Biocheck Elisa kit (USA) was utilized to estimate cardiac troponin-T (cTn-T) and cardiac troponin-I (cTn-I) levels in rats.

### Determination of cytokine level

Serum interleukin (IL)-6, IL-10, IL-1β, Tumor necrosis factor (TNF)-α, Transforming growth factor (TGF)-β (eBioscience), leptin (Ray biotech, USA) and adiponectin (Acrp30, R&D Systems) level was estimated by rat ELISA kit by using micro titer plate reader. The assay was performed as per the detailed instructions of the manufacturer.

### Hepatic and cardiac lipid content

The hepatic and cardiac TC and TG contents were determined by Wako kits (Wako Pure Chemical Industries, Ltd.). For the measurement of free fatty acid (FFA) content, tissues were homogenized in 200 µl of chloroform with 1% Triton X‑100. Fatty acids were extracted in the chloroform fraction and N2-dried to remove the chloroform. Then, the hepatic and cardiac tissue FFA contents were determined by FFA quantification kit (BioVision, Mountain View, CA, USA)^23^.

### Preparation of tissue extract

Liver and heart were isolated for preparation of tissue homogenate from all groups of animals for estimation of enzymatic and nonenzymatic antioxidant and oxidative stress parameters with protease inhibitor cocktail as per previously published procedure of Banerjee et al.^1^.

### Isolation of hepatocytes and cardiomyocytes

Hepatocytes and cardiomyocytes were isolated by previously published perfusion technique method by using collagenase type-II enzyme^24,25^.

### Flowcytometric analysis of reactive oxygen species (ROS)

Intracellular ROS was measured by DCFH-DA as per the directions of the manufacturer (Thermo Fisher Scientific, Waltham, MA, USA) by using BD FACS VERSE with excitation at 488 nm (blue filter). Finally the emission was collected by 527/32 nm band pass filter and data were analyzed by using FlowJo software^1^.

### Estimation of thiobarbituric acid reactive substances (TBARS) and nitric oxide production

Generation of TBARS as byproduct of lipid peroxidation was evaluated by TBA^26^. Data were expressed in nmoles of TBARS per milligram of protein using the molar extinction coefficient (1.56 × 10^5^ cm^2^/mM). Generation of nitric oxide (NO) was measured by Griess reaction^27^. Sodium nitrite standard curve was used to compute the presence of nitrite in the sample and expressed in µmole per milligram of protein**.**

### Estimation of superoxide dismutase (SOD)

NBT method was used to estimate SOD activity which depends on the hindrance of NBT decline by it^28^. The relative absorbance was then changed over into unit of SOD activity/mg of protein, where one unit of the activity of SOD was equal to the amount of SOD that produced half reduction in background rate of NBT decrease.

### Estimation of catalase (CAT)

Activity of CAT was determined by Beer’s method^29^ by the deterioration of H_2_O_2_ at 240 nm. The distinction in absorbance per unit time was used as an estimation of the activity of CAT. The values were communicated as U/mg of protein.

### Estimation of glutathione (GSH)

Elman method was used to estimate the level of GSH by using DTNB. The absorbance of decreased chromogen was pursued spectrophotometrically at 412 nm. GSH level was estimated by using a standard curve and values were expressed as nmoles/mg of protein^30^.

### Estimation of protein

Lowry method was utilized to estimate the protein content in tissue homogenate of liver and heart, isolated hepatocytes and cardiomyocytes by using BSA as standard^31^**.**

### Determination of mitochondrial membrane potential (MMP)

MMP was measured by using lipophilic dye DiOC6 according to the instructions manual (Thermo Fisher Scientific, Waltham, MA, USA) and analyzed by using BD FACS VERSE at 488 nm. Finally emission was collected at 527/32 nm band pass filter and the outcomes were investigated by using the FlowJo software^1^.

### Cell cycle study using propidium iodide

RNase-PI (ribonuclease with propidium iodide, Thermo Fisher Scientific, Waltham, MA, USA) was utilized for the analysis of cell cycle of hepatocytes and cardiomyocytes by previously published method of Banerjee A et al. (2020); BD VERSE was utilized for this measurement with excitation at 488 nm and outflow was collected by 585/40 nm band pass filter. Finally the percentages of cells in different phases were analyzed by using the FlowJo software^1^.

### Histopathological analysis and Oil red O staining

The liver and heart were fixed in 10% buffered formalin, processed, and embedded in paraffin for hematoxylin–eosin (H & E) staining and microscopic photographs were taken for analysis. Frozen samples prepared at the optimal cutting temperature were also being stained with Oil Red O. The microscope slides were analyzed by using compound microscope with camera attachment (Primo star model, Carl Zeiss Meditec, Dublin, CA) and photographs of the respective sections were taken.

### Immunohistochemistry

Immunohistochemistry was carried out on paraffin sections with anti-NF-kB (p65), cleaved caspase 3 antibodies. Briefly, xylene was used to deparaffinize the sections and followed by permeabilisation by treating with 0.1% Triton X100. Then unmasking of antigens was performed by heating the sections at 90 °C for 10 min in 10 mM citrate buffer, pH 6. After cooling at room temperature for 30 min each section was treated with the diluted primary antibodies overnight at 4 °C. After that, the sections were then washed with PBS and incubated with appropriate dilution of secondary antibody tagged with fluorescein isothiocyanate (FITC). Nuclei were stained by using DAPI. Fluorescent signals were viewed under a microscope (Olympus IX81). To observe any nuclear translocation of NF-kB (p65) and cleaved caspase 3, the colour of FITC was merged with the corresponding DAPI stained nuclei. Quantification of NF-kB and cleaved caspase 3 nuclear translocation was done by evaluating the color intensity using Image J software (version 2)^32^.

### Total RNA isolation and RT-PCR

Trizol reagent was used to isolate RNA from the isolated hepatocytes and cardiomyocytes as per the instruction of the suppliers (Gibco Laboratories, USA). ND-1000 spectrometer (Nanodrop Technologies, Wilmington, DE) was used to measure the concentrations of the isolated RNA at 260 nm wavelength. Single stranded complementary DNA (cDNA) was synthesized from total RNA by using cDNA reverse transcription kit with RNAase inhibitor (Applied Biosystems, USA). Then the produced cDNA was amplified using green master mix (Fermentas, USA) using the primers. Reverse transcriptase polymerase chain reaction (RT-PCR) was performed with Qiagen one-step RT-PCR kit (Qiagen, Germany) as per the manufacturer’s instructions. Forward and reverse gene specific primers for isolated hepatocytes iNOS^33^ product size 1,383 base pairs (forward 5′CGAGGAGGCTGCCCTGCAGACTGG3′ and reverse 5′CTGGGAGGAGCTGATGGAGTAGTA3′), eNOS^34^ product size 202 base pairs (forward 5′TGGGCAGCATCACCTACGATA3′ and reverse 5′GGAACCACTCCTTTTGATCGAGTTAT3′), TNF-α^35^ product size 592 base pairs (forward 5′ACGCTCTTCTGTCTACTG3′ and reverse 5′GGATGAACACGCCAGTCG3′), β-actin^35^ product size 477 base pairs (forward 5′CCTGCGTCTGGACCTGGCTG3′ and reverse 5′CTCAGGAGGAGCAATGATCT3′) and for isolated cardiomyocytes iNOS^36^ product size 576 base pairs (forward 5′GTGTTCCACCAGGAGATGTTG3′ and reverse 5′CTCCTGCCCACTGAGTTCGTC3′), eNOS^36^ product size 614 base pairs (forward 5′CCGGAATTCGAATACCAGCCTGATCCATGGAA3′ and reverse 5′GCCGGATCCTCCAGGAGGGTGTCCACCGCATG3′), TNF-α^36^ product size 546 base pairs (forward 5′CACGCTCTTCTGTCTACTGA3′ and reverse 3′GGACTCCGTGATGTCTAAGT5′), β-actin^37^ product size 138 base pairs (forward 5′CTCTGTGTGGATCGGTGGCT3′ and reverse 5′GCAGCTCAGTAACAGTCCGC3′) in a thermal cycler. The PCR products were analyzed by scanning densitometry (Image J, NIH) and values were normalized to quantity of β-actin and presented as % mRNA relative to control. The results were normalized to the levels obtained for the β-actin gene by taking a ratio of the value obtained for the gene of interest to that of β-actin^38^.

### Western blotting

In each lane equal amount of protein (50 μg) from isolated hepatocytes and cardiomyocytes were loaded for 12% sodium dodecyl sulphate–polyacrylamide gel electrophoresis (SDS-PAGE) and then electroblotted on polyvinylidene fluoride (PVDF) membrane (Millipore, Massachusetts, USA). 5% BSA solution in PBS-T was used for blocking the PVDF membrane at 4 °C overnight. The total protocol of western blotting was performed according to the previously published method^32^. After the blocking the membranes were subsequently incubated with the specific antibodies against the proteins such as Bcl2, Bax, cleaved caspase 3, cleaved caspase 9, PPAR-α, PPAR-γ, p21, p53 and GAPDH (each dilution 1:1,000) for 90 min at 37 °C; then PBS-T was used to wash the incubated membranes and then membranes were incubated at 37 °C with HRP-conjugated secondary antibodies (dilution 1:1,000) for 60 min. Finally, the protein-antibody complexes were observed by chemiluminescence (ECL system, Pierce). Protein expressions were normalized to that of endogenous control GAPDH.

### Annexin V APC staining for detection of apoptosis

The assay was performed according to the instruction of the manufacturer (Thermo Fisher Scientific, Waltham, MA, USA) and analysis was done in Flowjo software.

### Statistical analysis by StatsDirect 3.0 software

Data were expressed as Mean ± SE. To reveal whether the scores of different groups varied significantly, Kruskal–Wallis nonparametric analysis of variance (ANOVA) test was executed and to find out inter-group significance difference, Mann–Whitney *U* multiple comparison tests were executed to find the connection between the variables in the investigation. Statistical analysis was executed by using StatsDirect 3.0 software (United Kingdom). Differences were considered significant if *P* < 0.05^1^.

## Results

### HPTLC, GCMS analysis of EECGL and acute toxicity study of EECGL

**Figure 2:**
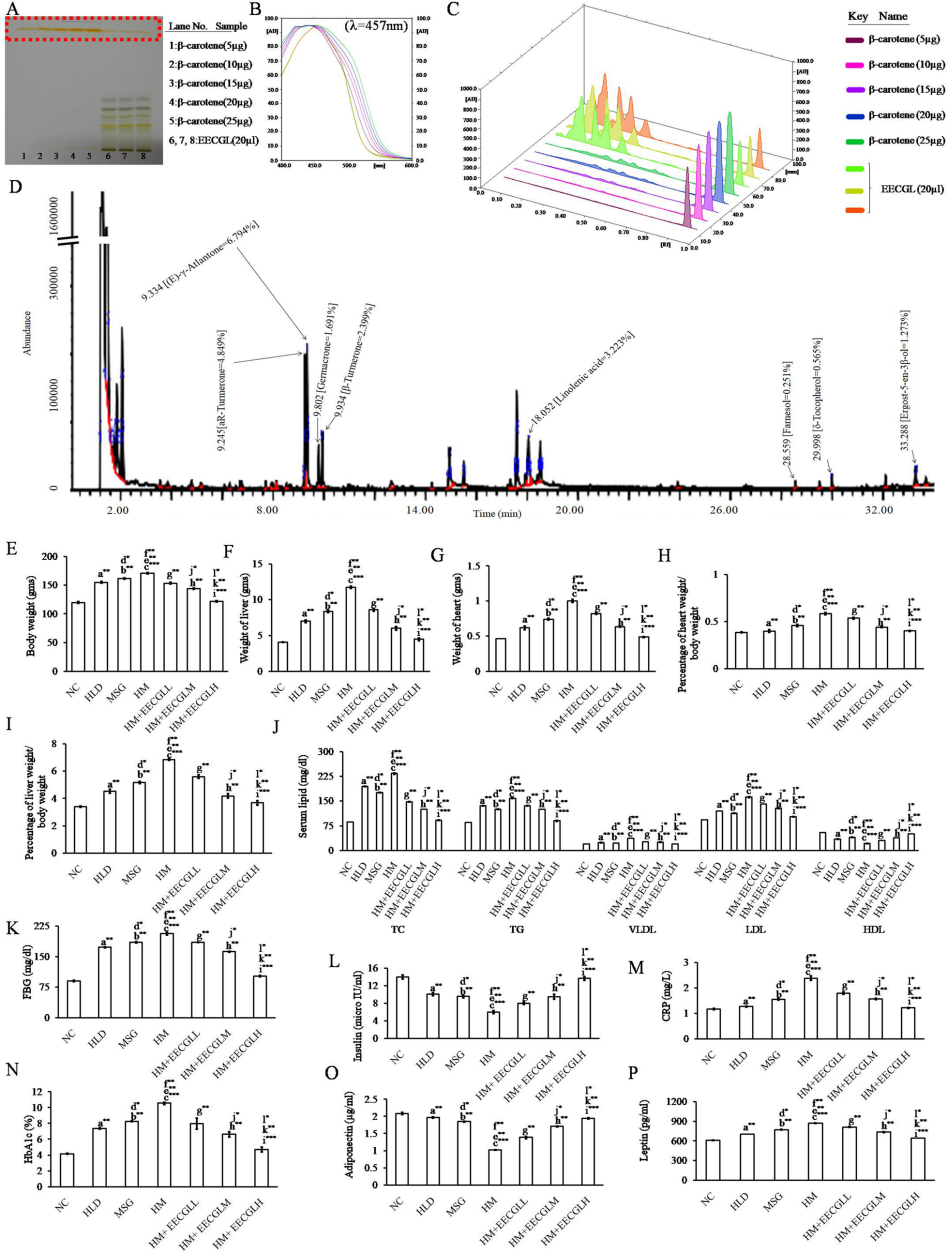
Chromatographic identification of compound(s) present in the EECGL and effect of EECGL on HM induced altered body weight, organ weight, serum lipid, glucose levels and insulin resistance in rats. (**A**) HPTLC fingerprint showed prominent band of β-carotene (from lane 6–8 marked by red colour) of EECGL with standard β-carotene (from lane 1–5, marked by red colour with increasing concentration of the standard). (**B**) Overlay spectra of the EECGL with five increasing concentration of β-carotene as a standard. (**C**) Densitogram represented five increasing concentration of β-carotene as a standard (β-carotene peak marked by plum, lilac, purple, dark blue, forest green colour) with that of the extracts obtained from EECGL (β-carotene peak marked by lime, olive, orange colour). (**D**) GCMS analysis and components present in the EECGL with their retention time (left side of respective compound) and total percentage (right side respective compound). Rats were feed with HLD, MSG, HM and HM + three increasing concentration of EECGL for 28 days with regular observation of body weight and after 28 days (**E**) average body weight, (**F**) liver weight, (**G**) heart weight were measured and (**H**) percentage of heart weight/body weight, (**I**) percentage of liver weight/body weight were calculated. The Bar diagram represented the (**J**) serum lipid levels, (**K**) FBG, (**L**) insulin, (**M**) CRP, (**N**) HbA1c, (**O**) adiponectin and (**P**) leptin of the control, treatment variables and the supplements. Statistical comparison by Kruskal–Wallis nonparametric ANOVA test [*P* < 0.05]. Significance level based on Mann–Whitney U multiple comparison test: a-NC vs. HLD, b-NC vs. MSG, c-NC vs. HM, d-HLD vs. MSG, e-HLD vs. HM, f-MSG vs. HM, g-HM vs. HM + EECGLL, h-HM vs. HM + EECGLM, i-HM vs. HM + EECGLH, j-HM + EECGLL vs. HM + EECGLM, k-HM + EECGLL vs. HM + EECGLH, l-HM + EECGLM vs. HM + EECGLH [*P < 0.05, **P < 0.01, ***P < 0.001].

Enormous numbers of known bioactive compounds were present in EECGL which was uncovered by HPTLC and GCMS study in the present investigation. Retention time and percentage (%) of the bioactive compounds present in EECGL were summarized in the Fig. 2A,D. HPTLC result showed that 100 g of *Coccinia grandis* leaves in dry powder form contains 60.47 mg of β-carotene (Fig. 2A–C) and GCMS analysis (Fig. 2D) showed different known active compounds present in EECGL, such as (E)-γ-Atlantone (6.794%), aR-Turmerone (4.849%), Linolenic acid (3.223%), β-Turmerone (2.399%), Germacron (1.691%), Ergost-5-en-3β-ol (1.273%), δ-Tocopherol (0.565%) and Farnesol (0.251%) respectively, which have different therapeutic values in various disorders as mentioned in the discussion part.

From acute toxicity study, it has been observed that EECGL was safe up to a dose of 5,000 mg/kg body weight orally. No toxicity or mortality reactions were observed after observing the behavior of the animals closely for the first 8 h and then an interval of every 4 h during the next 48 h.

### Ameliorative effect of EECGLs on HM induced changes on body weight, organ weight, percentage of organ weight/body weight, fasting blood glucose, HbA1c, insulin and serum lipid level

In the present study, it has been observed that a significant increase in body weight, organ weight, percentage of organ weight/body weight (*P* < 0.01) associated with significantly elevated levels of (*P* < 0.01) TC, TG, VLDL and LDL (Fig. 2E–J) with reduced level of (*P* < 0.01) HDL in HLD, MSG fed animal compare with the control (NC) group. But on the contrary it was maximum in HM fed rats (*P* < 0.001) compare with the control showed that high lipid diet in combination with MSG cause hyperlipidemic effect with increased (*P* < 0.001) fasting blood glucose (Fig. 2K) level (56.10%) and HbA1c (60.63%) level (Fig. 2N). Although the present study, did not observed any significant changes in daily food intake (Supplementary Fig. S1) among different group of animals. Furthermore, plasma insulin level (Fig. 2L) was significantly (*P* < 0.001) reduced (57.13%) in rats treated with HM and in both HLD, MSG group alone (*P* < 0.01) when contrasted with those of NC group. The most noticeable impact was observed in HM treated group as compared to the control group (*P* < 0.001) which was dose dependently reduced (*P* < 0.01) by the two different concentrations of the supplement EECGLs (EECGLL, EECGLM) but EECGLH showed the most significant ameliorative effect (*P* < 0.001). The serum lipid profile in the HM group were significantly higher (increased by 63.29% TC, 46.52% TG, 48.18% VLDL, 42.29% LDL and decreased by 60.82% HDL levels) than those in the NC group (*P* < 0.001), while three EECGLs intervention groups were significantly reverse the effects (*P* < 0.01) but EECGLH supplementation significantly (*P* < 0.001) decrease TC by 60.96%, TG by 43.07%, VLDL by 46.22% and LDL by 36.72% and enhance HDL by 57.78% compared with those in the HM group. Increasing EECGLs doses cause a further reduction in increased serum lipid levels such as TC, TG, LDL, and VLDL. In addition, HM group when treated with EECGLH, the fasting blood glucose (FBG) level and HbA1c level were significantly decreased (FBG, 50.72%, *P* < 0.001 and HbA1c, 55.51%, *P* < 0.001) on the other hand, insulin level was significantly (*P* < 0.001) elevated (56%). However, other two doses are efficacious but not up to the degree of efficacy exerted by EECGLH.

### Ameliorative effect of EECGLs on HM induced alterations on liver functional enzymes (ALT, AST and ALP) and cardiac marker enzymes (LDH, CK-MB, Troponin I and T)

**Figure 3:**
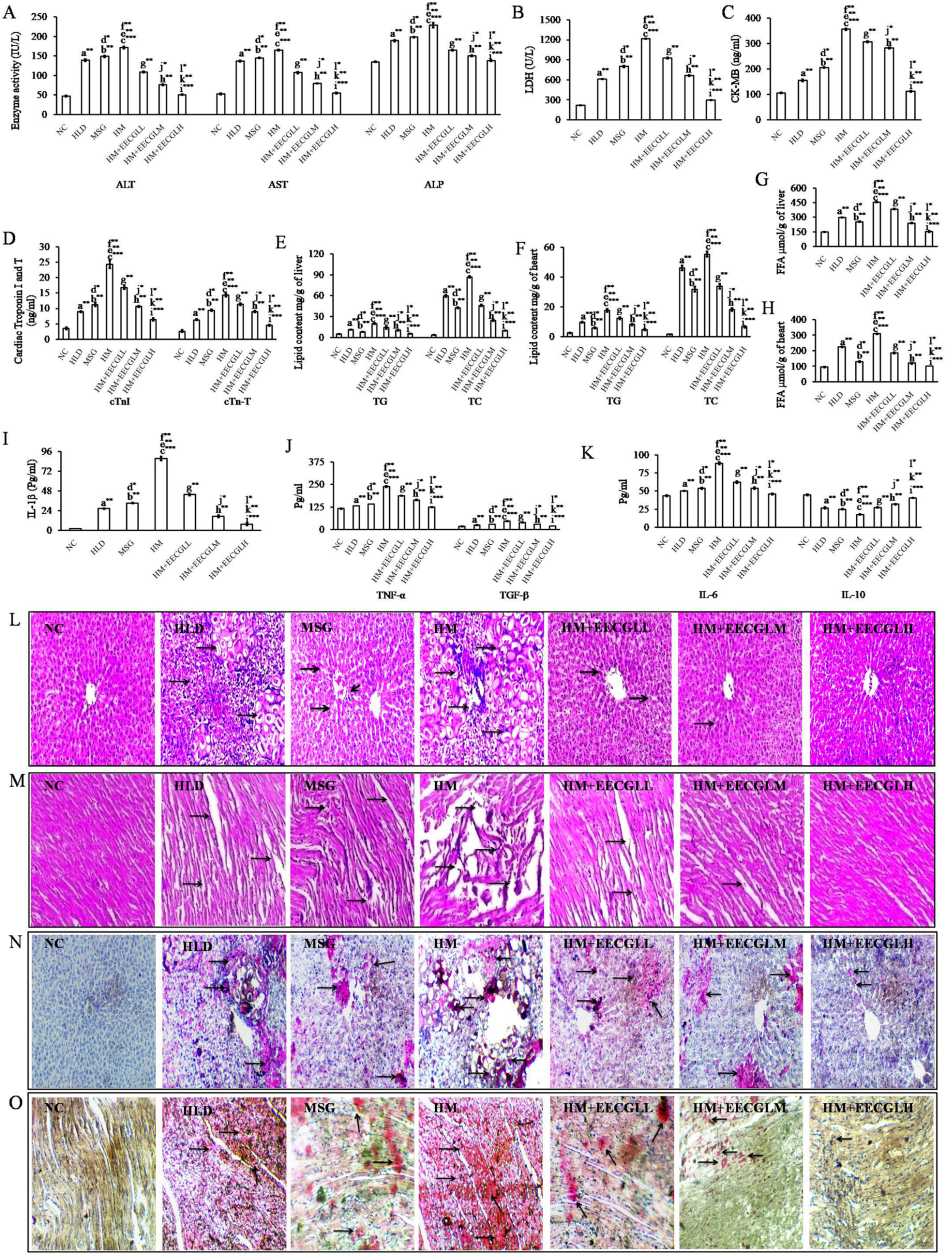
Effects of EECGL on hepatic and cardiac marker enzymes, lipid contents, serum inflammatory factors and tissue architecture. (**A**) hepatic marker enzymes, (**B**) LDH, (**C**) CK-MB, (**D**) cardiac troponin I and T, (**E**) hepatic lipid content, (**F**) cardiac lipid content, (**G**) hepatic FFA, (**H**) cardiac FFA, (**I**) IL-1β, (**J**) TNF-α and TGF-β, (**K**) IL-6 and IL-10. The liver and heart pathologies in the different groups of HLD, MSG, HM and HM + EECGL fed rats were observed. Bright field microscopy images of liver, heart tissue by (**L**,**M**) Hematoxylin and eosin (HE) staining (20 ×) and (**N**,**O**) Oil Red O staining (20 ×). Significance level based on Mann–Whitney U multiple comparison test: a-NC vs. HLD, b-NC vs. MSG, c-NC vs. HM, d-HLD vs. MSG, e-HLD vs. HM, f-MSG vs. HM, g-HM vs. HM + EECGLL, h-HM vs. HM + EECGLM, i-HM vs. HM + EECGLH, j-HM + EECGLL vs. HM + EECGLM, k-HM + EECGLL vs. HM + EECGLH, l-HM + EECGLM vs. HM + EECGLH [*P < 0.05, **P < 0.01, ***P < 0.001].

Liver function tests were used to evaluate liver functional status after HM administration. After the experimental period was over, ALT, AST and ALP levels (Fig. 3A) were significantly higher in the HM group with compare to the NC group (*P* < 0.001). The serum ALT, AST and ALP levels were significantly increased (*P* < 0.001) by 72.51%, 68.01% and 40.98%, respectively, in the HM group compared with the NC group. However, the ALT, AST and ALP levels in the three EECGLs intervention groups were decreased to varying degrees compared with those in the HM group. After EECGLs intervention, the serum ALT, AST and ALP levels exhibited downward trends compared with those in the HM group. The ALT, AST and ALP levels were decreased by 70.46%, 66.86%, and 39.67%, respectively, in the EECGLH group compared with the HM group. In particular, the ALT activity was significantly lower (70.46%) in the EECGLH group than in the HM group (*P* < 0.001). Simultaneously, cardiac marker LDH, CK-MB, Troponin I and T (Fig. 3B–D respectively) were significantly increased (*P* < 0.001) by 82.13%, 70.29%, 85.61% and 81.69%, respectively, in the HM group with compare to the NC group. After EECGLs supplementation LDH, CK-MB, Troponin I and T were significantly decreased by 75.51%, 68.53%, 73.65% and 68.52%, respectively, in the EECGLH group (*P* < 0.001) compared with the HM group. Furthermore, rests of the two doses are effective but not up to the degree of affectivity exerted by EECGLH.

### Effects of EECGLs on high-sensitivity C-reactive protein (hs-CRP) and serum inflammatory factors

The increase level of hs-CRP, which is known to be a risk factor of CVD, is elevated in NAFLD patients, particularly in those with NASH. Results of our present study showed that the level of hs-CRP (Fig. 2M) was significantly higher (*P* < 0.001) in the HM group compared with the NC group (increased by 50.75%), whereas it was successfully controlled by the three different concentrations of EECGLs treatment in contrast to the HM group (decreased by 24.70%, 34.27%, 48.74%) and remained close to the level in the NC group, which further demonstrated the anti-inflammatory potential of EECGLs mainly EECGLH (*P* < 0.001). The serum IL-1β, TNF-α, TGF-β, IL-6 (Fig. 3I–K respectively) and leptin (Fig. 2P) levels increased by 97.48%, 51.31%, 63.64%, 50.83% and 30.20%, respectively, in the HM group compared with the NC group; on the contrary the IL-10 (Fig. 3K) and adiponectin (Fig. 2O) levels decreased by 60.11% and 51.10% (*P* < 0.001) respectively. The serum IL-6, IL-1β, TNF-α, TGF-β and leptin levels decreased significantly (*P* < 0.001) by 47.86%, 91.71%, 47.72%, 56.55% and 26.06% respectively with increased levels of adiponectin and IL-10 by 47.55% and 56.13%, respectively, in the EECGLH supplementary group compared with the HM group. The effects of low, medium dose of EECGL (*P* < 0.01) and high dose of EECGL (*P* < 0.001) on cytokine levels significantly differ from the effects of HM. On the other hand, the other two doses of EECGL are ameliorative on HM induced alterations but not up to the degree of amelioration exerted by EECGLH.

### Effects of EECGLs on HM induced changes in rat hepatic and cardiac lipid content

Hepatic and cardiac lipid content (Fig. 3E–H) estimation was performed to assess the effects of EECGLs on lipid accumulation in the liver and heart. Figure 3 shows that hepatic and heart lipid content was significantly higher (*P* < 0.01) in the HLD (hepatic TC increased by 93.73%, TG increased by 55.23%, FFA increased by 50.23%; Cardiac TC increased by 96.45%, TG increased by 73.33%, FFA increased by 57.73%), in the MSG (hepatic TC increased by 91.27%, TG increased by 36.37%, FFA increased by 41.65%; Cardiac TC increased by 94.86%, TG increased by 55.74%, FFA increased by 27.22%) and much higher (*P* < 0.001) in the HM group (hepatic TC increased by 95.73%, TG increased by 75%, FFA increased by 67.59%; Cardiac TC increased by 97.05%, TG increased by 85.25%, FFA increased by 69.44%) than in the NC group. While the EECGLH supplementation cause significant decrease of hepatic TC, TG, and FFA levels by 88.64%, 71.16% and 66.47%, respectively, with simultaneous significant decrease of cardiac TC, TG, and FFA levels by 88%, 73.75% and 67.47%, respectively, as compared with the HM group (*P* < 0.001). Further increase in the dose of EECGL did not exert more pronounced lipid-lowering effect.

### EECGLs prevent fatty liver escalated hepatic damage and cardiac damage induced by co administration of high lipid diet and monosodium glutamate

**Figure 4:**
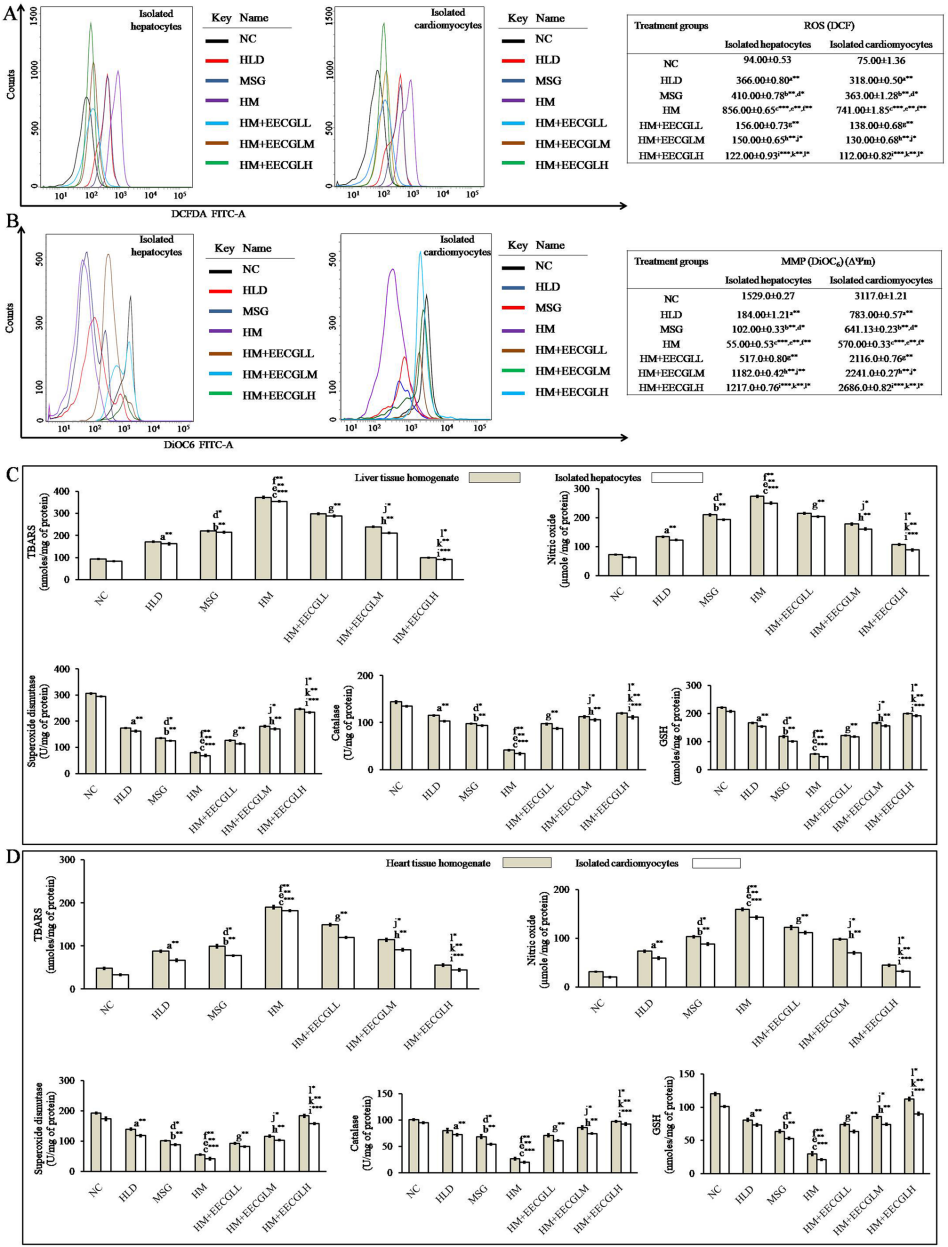
Effects of EECGL on hepatic and cardiac oxidative stress and mitochondrial membrane potential. (**A**) Overlaid histogram plot of ROS generation in control, HLD, MSG, HM and HM + EECGL treated group. The movement of histogram towards right indicated the higher ROS generation. DCF intensity was taken along the X axis and cell count was taken along the Y-axis. Different colours of histogram represented the ROS generation in different experimental groups. On the right side of the overlay the table represented the data mean fluorescence intensity of DCF. (**B**) Overlaid histogram plot of MMP in control, HLD, MSG, HM and HM + EECGL treated group. The movement of histogram towards left indicated the loss MMP. DiOC6 intensity was taken along the X axis and cell count was taken along the Y-axis. Different colours of histogram represented the MMP in different experimental groups. On the right side of the overlay the table represented the data mean fluorescence intensity of DiOC6. (**C**) Bar diagram represented the TBARS, NO, SOD, CAT, GSH level in control, HLD, MSG, HM and HM + EECGL treated groups from liver tissue homogenate and isolated hepatocytes, respectively. (**D**) Bar diagram represented the TBARS, NO, SOD, CAT, GSH level in control, HLD, MSG, HM and HM + EECGL treated groups from heart tissue homogenate and isolated cardiomyocytes, respectively. Significance level based on Mann–Whitney U multiple comparison test: a-NC vs. HLD, b-NC vs. MSG, c-NC vs. HM, d-HLD vs. MSG, e-HLD vs. HM, f-MSG vs. HM, g-HM vs. HM + EECGLL, h-HM vs. HM + EECGLM, i-HM vs. HM + EECGLH, j-HM + EECGLL vs. HM + EECGLM, k-HM + EECGLL vs. HM + EECGLH, l-HM + EECGLM vs. HM + EECGLH [*P < 0.05, **P < 0.01, ***P < 0.001].

From the histopathological study by using H & E stain (Fig. 3L), it was revealed that hepatic tissue sections in HM fed rats showed morphological changes as compared to control group (NC). In case of NC group there was no pathological changes with normal architecture of liver was observed. As HLD and MSG independently generate ROS^6^ and the present study showed that HM induced reactive oxygen species generation and subsequent oxidative stress (Fig. 4), may be the plausible cause for liver damage in this experimental set up. Hepatocellular cytoplasmic vacuolization with prominent fat deposition was identified in HLD fed rats associated with inflammations; on the other hand, moderate degree of atrophy, inflammatory as well as degenerating hepatocytes, mild necrotic cells and proliferation of hepatocytes were observed in MSG fed group. But, in HM fed group the degree of severity was maximized as contrast to the HLD and MSG group. Typical characteristics of liver damage with disturbed normal radiating pattern, damaged cell membranes, ruptured central vein and endothelial lining, fat depositions, damaged hepatic cords and sinusoidal network, severe atrophy of hepatocytes, disrupted nuclei of hepatocyte indicates hepatic inflammation and damage of hepatic tissue. On the contrary, gradual improvements were uncovered by EECGL supplementation. In HM + EECGLL and HM + EECGLM supplementary group gradual improvement of normal radiating pattern of the central vein, cell plates and very few numbers of lipid droplets or reduced diameter of the lipid droplets, were found. However, EECGLL and EECGLM exerted moderate degree of protection against HM induced alterations but not up to the degree of protection exerted by EECGLH where the boundaries between hepatocytes were more normal and it was very easy to identify its normal radiating pattern and lobular structures.

The H & E staining of cardiac tissue section (Fig. 3M) showed that no pathological changes was observed in the NC group of animals; but intermittent loss of fiber of cardiac muscle with extreme aggravation, lipid accumulation in between cardiac muscle fibers were observed in HLD fed animals. The stained cardiac tissue from the MSG group showed moderate degree of damage in cardiac tissue architecture with central cellular infiltration of muscle fiber associated with mild degree of inflammation and loss of muscle fibers. But, in HM fed group the degree of cardiac tissue damage was as much as observed in HLD and MSG fed group. H & E staining of cardiac tissue in HM group showed occasional loss of muscle fiber with inflammation, intramyocardial lipid accumulation and severe necrotic changes of karyolysis. These further indicate hyalinization of muscle fibers with contrast to NC group. However, the supplementation significantly blunted this deleterious effect of HM induced cardiac damage in dose dependent manner by EECGLs. In the supplemented groups (HM + EECGLs) mild necrosis, mild swelling of cardiomyocytes and focal cardiac muscle fibers with minimal damage of cardiac tissue was identified by H & E staining procedure. Gradual improvements of cardiac muscle as well as cardiomyocytes were observed and revealed regeneration of cardiac muscle cells with prominent nucleoli, absence of intramyocardial lipid in the HM + EECGLs supplemented groups. In addition, EECGLL and EECGLM showed moderate degree of improvement against HM induced alterations but not up to the degree of improvement exerted by EECGLH.

Further for more confirmation about the lipid accumulation in hepatic (Fig. 3N) and cardiac (Fig. 3O) tissue we have checked it through Oil Red O staining procedure. In case of NC group, no lipid accumulation or very small amount of lipid accumulation was observed (with small red area); in HM fed rats an increased number of lipid droplets with red area representing the presence of fats but in HLD, MSG fed animals the lipid accumulation were not as much as observed the HM group. The degrees of fat depositions were reduced in number in all HM + EECGLs supplementation with contrast to the HM group. On the other hand, EECGLL and EECGLM indicated moderate degree of protection against HM induced fat depositions but not up to the degree of improvement revealed by EECGLH. These results strongly suggest that EECGLs exerts certain preventive effects on HM-induced NAFLD associated with hepatic and cardiac injury which further leads to coronary heart disease.

### Effects of EECGLs on HM induced alterations in oxidative stress parameters in isolated hepatocytes and cardiomyocytes as well as liver and heart tissue homogenate by the generation of intracellular reactive oxygen species (ROS) and disruption of mitochondrial membrane potential (MMP)

Intracellular ROS generation in isolated hepatocytes and cardiomyocytes were measured by flow cytometry using H_2_DCFDA (Fig. 4A). In isolated hepatocytes, HLD (366.00 ± 0.80 DCF fluorescence intensity in arbitrary unit) and MSG group (410.00 ± 0.78 DCF fluorescence intensity in arbitrary unit) showed a significant (*P* < 0.01) increase in ROS generation with respect to control (94.00 ± 0.53) group (Fig. 4A). Moreover, combined treatment of HLD and MSG, i.e., HM group showed the deleterious effect (*P* < 0.001) in the intracellular ROS generation (856.00 ± 0.65) as compared to the control (9.10 fold increase). The generation of ROS in HM + EECGLs group were in decreasing order like HM + EECGLL 156.00 ± 0.73, HM + EECGLM 150.00 ± 0.65 (*P* < 0.01) and HM + EECGLH 122.00 ± 0.93 (*P* < 0.001) compared to the HM (856.00 ± 0.65) group. In isolated cardiomyocytes, the data are well in line with the results obtained in hepatocytes. HLD (318.00 ± 0.50 DCF fluorescence intensity in arbitrary unit) and MSG group (363.00 ± 1.28 DCF fluorescence intensity in arbitrary unit) showed a significant (*P* < 0.01) increase in ROS generation with respect to control (75.00 ± 1.36) group (Fig. 4A). Moreover, in cardiomyocytes combined treatment of HLD and MSG (HM group) showed the deleterious effect (*P* < 0.001) in the intracellular ROS generation (741.00 ± 1.85). A significant increase in intracellular ROS production by 9.88 fold compared to control (NC) was found when treatment with HM. The formation of ROS in HM + EECGLs group were in decreasing order like HM + EECGLL 138.00 ± 0.68, HM + EECGLM 130.00 ± 0.68 (*P* < 0.01) and HM + EECGLH 112.00 ± 0.82 (*P* < 0.001) compared to the cells treated with HM (741.00 ± 1.85). In the both isolated hepatocytes and cardiomyocytes EECGLH dose showed the maximum inhibitory (*P* < 0.001) effect of ROS generation by 7.01 and 6.62 folds with compare to the HM group.

Measurement of the mitochondrial membrane potential by use of the cationic dye DiOC_6_ in proportion to ΔΨm (Fig. 4B), showed that HLD (in isolated hepatocytes 184.00 ± 1.21 and in isolated cardiomyocytes 783.00 ± 0.57) or MSG (in isolated hepatocytes 102.00 ± 0.33 and in isolated cardiomyocytes 641.13 ± 0.23) or in combination of both affects maximally on mitochondrial permeability transition (in isolated hepatocytes 55.00 ± 0.53 and in isolated cardiomyocytes 570.00 ± 0.33). HLD and MSG treated groups significantly depolarized the mitochondrial membrane potential (*P* < 0.01) but when treated with both MSG and HLD (HM group) showed the maximum transition of mitochondrial permeability (*P* < 0.001). When ΔΨm decreases, DiOC_6_ aggregates depart from mitochondria and change to DiOC_6_ monomers (488 nm emission; blue color). The cells lost their potential after treatments were designated as apoptotic cells and rest designated as viable cells. Thus, mitochondrial damage preceded the induction of apoptosis indicating a role in HM mediated cytotoxicity (*P* < 0.001). But, these effects were significantly blunted (EECGLL, EECGLM: *P* < 0.01; EECGLH: *P* < 0.001) when HM treatment group were supplemented with different concentration of EECGLs (in isolated hepatocytes 517.0 ± 0.80, 1,182.0 ± 0.42, 1,217.0 ± 0.76 and in isolated cardiomyocytes 2,116.0 ± 0.76, 2,241.0 ± 0.27, 2,686.0 ± 0.82).

Figure 4C,D, showed the effects of EECGLs on in vivo antioxidant activity. The superoxide dismutase (SOD) significantly decreased by 73.73% in liver homogenate, 76.46% in isolated hepatocytes, 71.11% in heart homogenate and 75.60% in isolated cardiomyocytes in the HM group compared with the NC group, and these differences were all statistically significant (*P* < 0.001). The catalase (CAT) activities significantly decreased by 70.80% in liver homogenate, 74.82% in isolated hepatocytes and 73.85% in heart homogenate and 79.07% in isolated cardiomyocytes in the HM group compared with the NC group, and these differences were all statistically significant (*P* < 0.001). The glutathione content (GSH) significantly decreased by 74.28% in liver homogenate, 77.75% in isolated hepatocytes, 75.13% in heart homogenate and 79.18% in isolated cardiomyocytes in the HM group compared with the NC group, and these differences were all significant (*P* < 0.001). The lipid peroxidation (TBARS) level in liver homogenate and isolated hepatocytes was increased by 74.84%, 76.49% and in heart homogenate and isolated cardiomyocytes, it was increased by 74.75%, 82.01%, respectively, in the HM group compared with the NC group, and these differences were all significant (*P* < 0.001). The nitric oxide (NO) level in liver homogenate and isolated hepatocytes was increased 73.26%, 74.76% and in isolated cardiomyocytes it was increased by 80.09%, 85.56% respectively, in the HM group compared with the NC group, and these differences were all statistically significant (*P* < 0.001). A significant increase (*P* < 0.01) in SOD activity, CAT activity and GSH content were observed in HM + EECGLL, HM + EECGLM supplemented groups when compared with HM group, but the most significant increase (*P* < 0.001) was uncovered in HM + EECGLH supplementation. Simultaneously, TBARS and NO levels in HM + EECGLL, HM + EECGLM intervention groups showed significant decrease (*P* < 0.01) level with compare to the HM group. But, the degree of this ameliorative effect was not as much as the HM + EECGLH (*P* < 0.001) fed group showed.

### Effect of EECGLs on HM induced alteration of cell cycle progression

**Figure 5:**
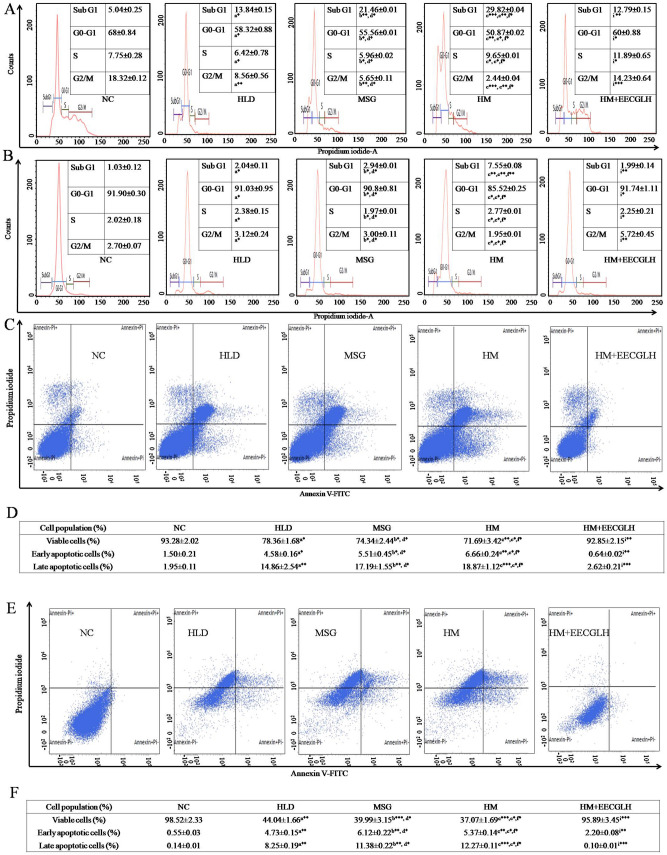
Determination of cell cycle progression by RNase PI and apoptosis by Annexin-FITC and PI. On the basis of size (FSC-H) and granularity (SSC-H) selected singlet population of (**A**) hepatocytes and (**B**) cardiomyocytes population was plotted. Graphs represented the distribution of cells in different phases of cell cycle. The first peak in all graphs represented the subG1 population, second peak represented the G0–G1 and third peak represented the G2/M population. The valley between these G0–G1 and G2/M peaks represented the S phase population. Percentage (%) of cells in different phases of cell cycle was represented in the respective figures. After completion of the experiment (**C**) hepatocytes and (**E**) cardiomyocytes from NC, HLD, MSG, HM and HM + EECGLH groups were stained by Annexin-FITC and PI. The Q1 quadrant represented the viable cell populations which was the maximum in NC group. Q2 quadrant represented the early apoptotic cell populations with FITC stain. The Q3 quadrant represented the late apoptotic cell population (dual stain positive cells). Intensity of FITC in FL1-H (FITC) channel was taken along the X-axis and FL2-H channel (PI) was taken along the Y-axis. Table represented the viable, early apoptotic and late apoptotic cell populations in control, HLD, MSG, HM and HM + EECGLH treated groups of the (**D**) hepatocytes and (**F**) cardiomyocytes. Significance level based on Mann–Whitney U multiple comparison test: a-NC vs. HLD, b-NC vs. MSG, c-NC vs. HM, d-HLD vs. MSG, e-HLD vs. HM, f-MSG vs. HM, i-HM vs. HM + EECGLH [*P < 0.05, **P < 0.01, ***P < 0.001].

Above data showed EECGLH possesses most significant effect with compare to the HM (*P* < 0.001), henceforth we have chosen only EECGLH group for the analysis of cell cycle progression (Fig. 5A,B). Single cell population was chosen to examine the cell cycle progression. The NC group of isolated hepatocytes (Fig. 5A) showed 5.04 ± 0.25% cell population in Sub G1 phase, 68 ± 0.84% cell population in G0–G1 phase, 7.75 ± 0.28% cell population in S phase and 18.32 ± 0.12% cell population in G2/M phase. HLD group showed that 13.84 ± 0.15% cell population in Sub G1 phase (*P* < 0.05), 58.32 ± 0.88% cell population in G0–G1 phase (*P* < 0.05), 6.42 ± 0.78% cell population in S phase (*P* < 0.05), 8.56 ± 0.56% cell population in G2/M phase (*P* < 0.01). MSG group showed that 21.46 ± 0.01% cell population in Sub G1 phase (*P* < 0.01), 55.56 ± 0.01% cell population in G0–G1 phase (*P* < 0.05), 5.96 ± 0.02% cell population in S phase (*P* < 0.05), 5.65 ± 0.11% cell population in G2/M phase (*P* < 0.01). HM group showed a significantly high cell population (29.82 ± 0.04%) in Sub G1 phase (*P* < 0.001), low cell population (50.87 ± 0.02%) in G0–G1 phase (*P* < 0.01) with 9.65 ± 0.01% cell population in S phase (*P* < 0.05) and 2.44 + 0.04% cell population in G2/M phase (*P* < 0.001). But, HM + EECGLH showed the significant effect on cell cycle progression study like 12.79 ± 0.15% cell population in Sub G1 phase (*P* < 0.01), 60.00 ± 0.88% cell population in G0–G1 phase (*P* < 0.05), 11.89 ± 0.65% cell population in S phase (*P* < 0.05), 14.23 ± 0.64% cell population in G2/M phase (*P* < 0.001).

Furthermore, the control group of isolated cardiomyocytes (Fig. 5B) showed 1.03 ± 0.12% cell population in Sub G1 phase, 91.90 ± 0.30% cell population in G0–G1 phase, 2.02 ± 0.18% cell population in S phase and 2.70 ± 0.07% population in G2/M phase. HLD group showed that 2.04 ± 0.11% cell population in Sub G1 phase (*P* < 0.05), 91.03 ± 0.95% cell population in G0–G1 phase (*P* < 0.05), 2.38 ± 0.15% cell population in S phase (*P* < 0.05), 3.12 ± 0.24% cell population in G2/M phase (*P* < 0.05). MSG group showed that 2.94 ± 0.01% cell population in Sub G1 phase (*P* < 0.05), 90.08 ± 0.81% cell population in G0–G1 phase (*P* < 0.05), 1.97 ± 0.01% cell population in S phase (*P* < 0.05), 3.00 ± 0.11% cell population in G2/M phase (*P* < 0.05). HM group showed a significantly high cell population (7.55 ± 0.08%) in Sub G1 phase (*P* < 0.01), low cell population (85.52 ± 0.25%) population in G0–G1 phase (*P* < 0.05) with 2.77 ± 0.01% cell population in S phase (*P* < 0.05) and 1.95 + 0.01% cell population in G2/M phase (*P* < 0.05). But, HM + EECGLH showed the significant effect on cell cycle progression study like 1.99 ± 0.14% cell population in Sub G1 phase (*P* < 0.01), 91.74 ± 1.11% cell population in G0–G1 phase (*P* < 0.05), 2.25 ± 0.21% cell population in S phase (*P* < 0.05), 5.72 ± 0.45% cell population in G2/M phase (*P* < 0.01).

### HM induced apoptosis measured by Annexin V/PI staining in isolated hepatocytes and cardiomyocytes

Apoptosis assay was conducted by Annexin V and Propidium iodide (PI) dye for staining at the cellular level using flow cytometry. Here we have chosen the three variables HLD, MSG, HM as treatment group and the most significant dose of EECGL (as per previous data and significant level), EECGLH as supplemented group. Flow cytometry analysis indicates significant increased percentage of early apoptotic cells (in isolated hepatocytes HLD, MSG: *P* < 0.05 respectively, HM: *P* < 0.01; in isolated cardiomyocytes HLD, MSG, HM: *P* < 0.01 respectively), late apoptotic cells (in isolated hepatocytes HLD, MSG: *P* < 0.01 respectively, HM: *P* < 0.001; in isolated cardiomyocytes HLD, MSG: *P* < 0.01; HM: *P* < 0.001 respectively) and significantly decreased percentage of viable cells in both isolated hepatocytes (Fig. 5C,D) (HLD, MSG, *P* < 0.05; HM, *P* < 0.01) and cardiomyocytes (Fig. 5E,F) (HLD: *P* < 0.01; MSG, HM: *P* < 0.001 respectively) of those treated with HM as compared to control and the percentages of apoptotic cells were significantly reduced in the most effective supplementary EECGLH fed group (for isolated hepatocytes late apoptotic cells *P* < 0.001, early apoptotic cells *P* < 0.01 and for isolated cardiomyocytes late apoptotic cells *P* < 0.001, early apoptotic cells *P* < 0.01 with compare to the HM) and percentage of viable cells were significantly increased (for isolated hepatocytes: *P* < 0.01 and for isolated cardiomyocytes: *P* < 0.001) with the supplementation of EECGLH.

### HM induced nuclear translocation of NF-kB (p65) and cleaved caspase 3 by immunohistochemistry in hepatic and cardiac tissue were alleviated by EECGLH

**Figure 6:**
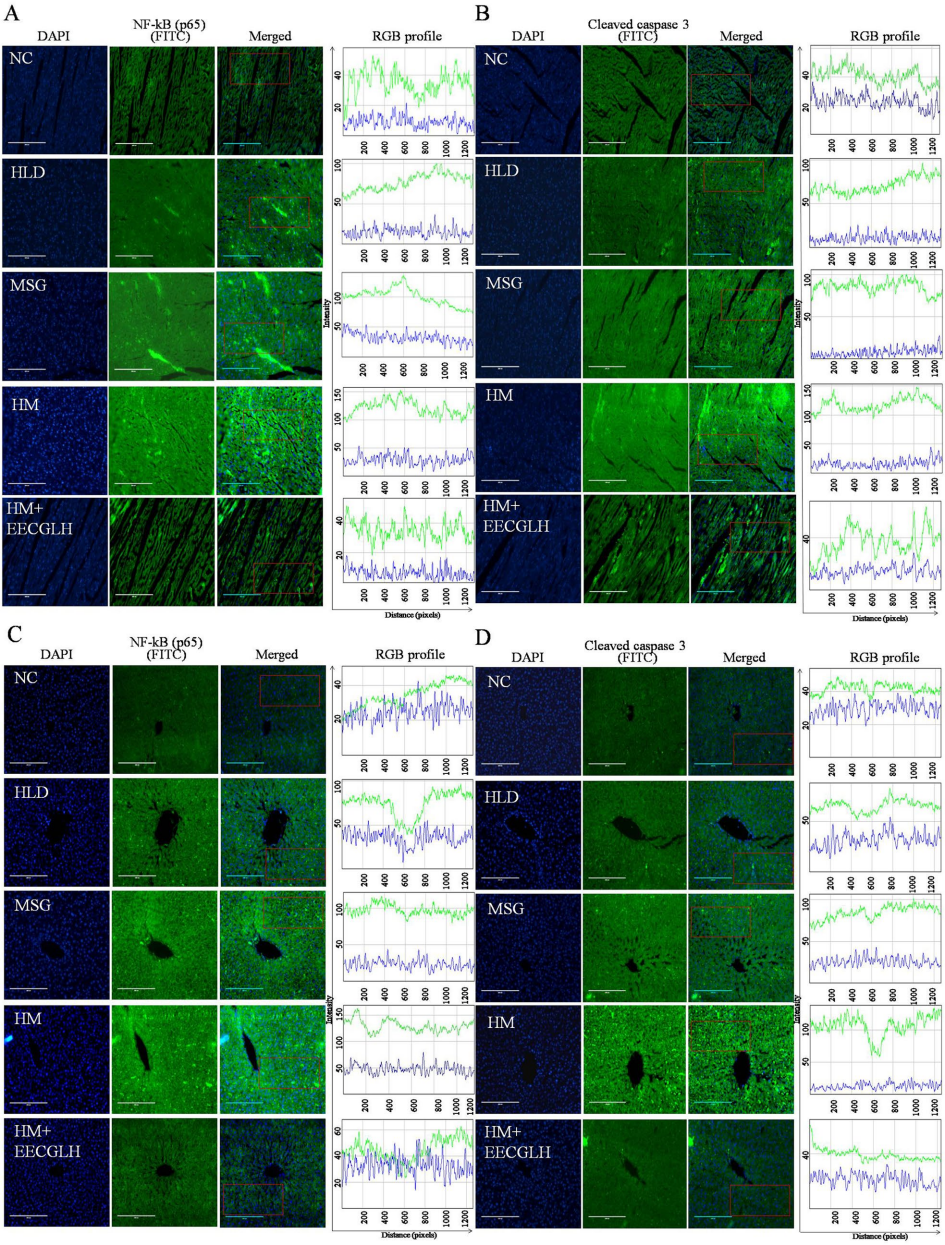
Nuclear translocation of nuclear factor kappa B (p65) and cleaved caspase 3 by immunohistochemistry of control, HLD, MSG, HM and HM + EECGLH treated groups of liver and heart tissue. Nuclear translocation of NF-kB (p65) of (**A**) heart tissue section and (**C**) liver tissue section were represented. Nuclear translocation of cleaved caspase 3 of (**B**) heart tissue section and (**D**) liver tissue section were represented. The nuclei were stained by DAPI which appeared blue and NF-kB (p65) or cleaved caspase 3 was stained by FITC tagged secondary antibody which appeared green. The merged images showed the infiltration of green colour into the blue region which indicated the nuclear translocation of NF-kB (p65) or cleaved caspase 3. NF-kB (p65) and cleaved caspase 3 positive nuclei were estimated from the selected region marked by red colour. The intensity of DAPI and FITC was plotted using ImageJ software (NIH Image J system, Bethesda, MD) for quantification of fluorescence intensity. The intensity (in arbitrary unit) was taken along the Y-axis and distance (in pixel) was plotted along the X-axis. For control, HLD, MSG, HM and HM + EECGLH groups the green line indicated the intensity of FITC and blue line indicated the intensity of DAPI.

**Figure 7:**
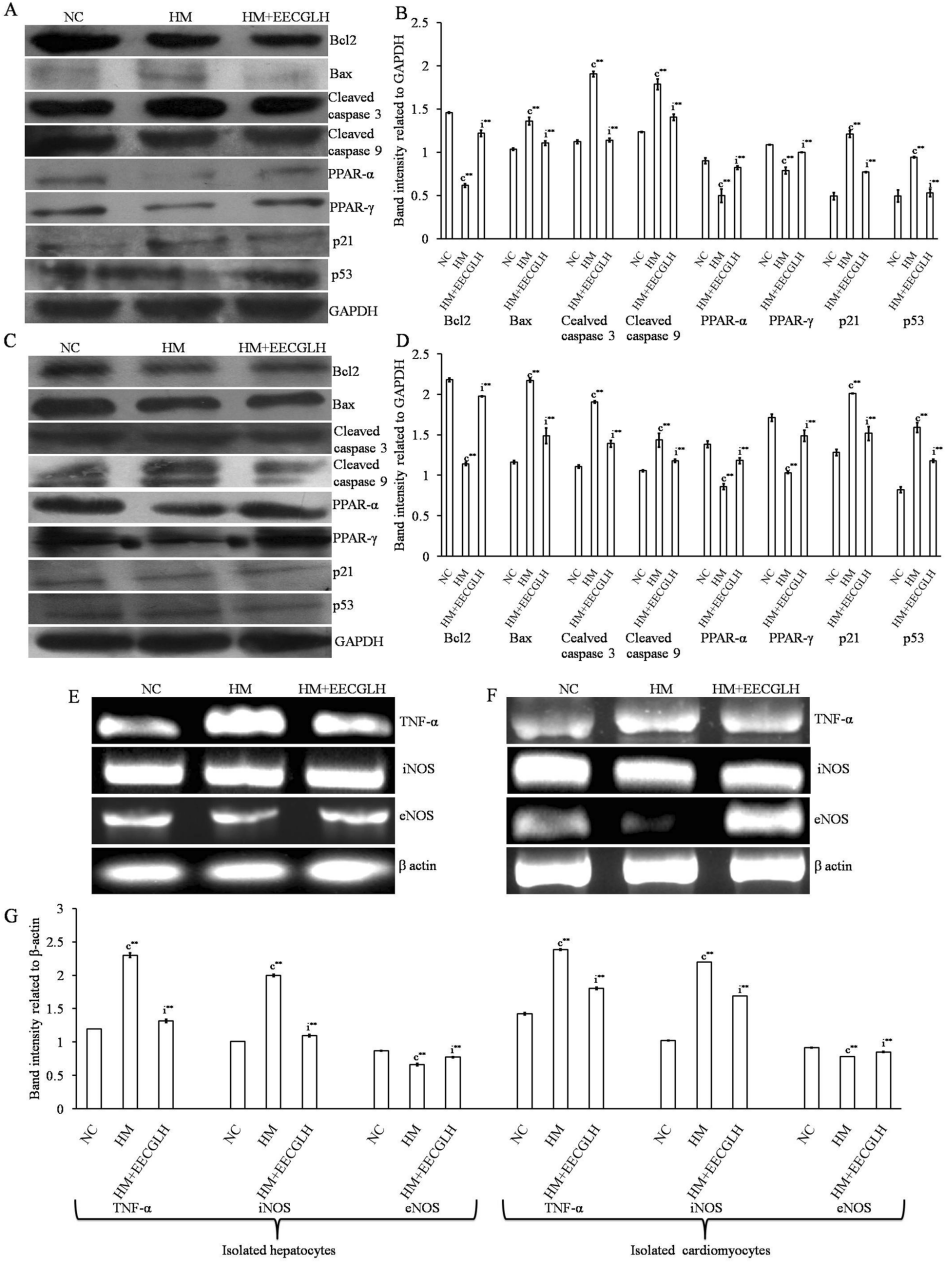
Protein expression in hepatocytes and cardiomyocytes in control, HM and HM + EECGLH group. Effects of EECGLH on HM induced alerted protein expressions of (**A**,**B**) hepatocytes and (**C**,**D**) cardiomyocytes with control groups with densitometric analysis some cell cycle regulator protein like p53 and p21, some transcription factors like PPAR-α and PPAR-γ, apoptosis related protein Bcl2, Bax, cleaved caspase 3 and 9 with an endogenous control GAPDH were represented. Gene expression in hepatocytes and cardiomyocytes in control, HM and HM + EECGLH group. Effects of EECGLH on HM induced alerted mRNA expression levels of (**E**) hepatocytes, (**F**) cardiomyocytes with control groups were represented and (**G**) bar diagram represented the densitometric analysis of TNF-α, iNOS, and eNOS with β-actin as endogenous control. Significance level based on Mann–Whitney U multiple comparison test: c-NC vs. HM, i-HM vs. HM + EECGLH [**P < 0.01].

Here, we have chosen only the HLD, MSG, HM group and HM + EECGLH (most effective group revealed by the statistical analysis with compare to the HM). To confirm the inflammatory development and apoptosis mediated by NF-kB (p65) and cleaved caspase 3, immunofluorescence method was adopted (Fig. 6). HM caused nuclear translocation of NF-kB (p65) and cleaved caspase 3; whereas NF-kB and cleaved caspase 3 remained localized in the cytoplasm of hepatocytes and cardiomyocytes in control and HM + EECGLH treated group. In case of control (NC) group, 3.33% nuclei were NF-kB positive**,** 5.17% nuclei were cleaved caspase 3 positive within the selected region of liver section and 3.70% nuclei were NF-kB positive**,** 6% nuclei were cleaved caspase 3 positive within the selected region of heart section; in HLD group, 30% nuclei were NF-kB positive**,** 12.20% nuclei were cleaved caspase 3 positive within the selected region of liver section and 29.63% nuclei were NF-kB positive**,** 15.38% nuclei were cleaved caspase 3 positive within the selected region of heart section; in MSG group showed moderate translocation of NF-kB and cleaved caspase 3 (37.5% nuclei were NF-kB positive**,** 16.67% nuclei were cleaved caspase 3 positive within the selected region of liver section and 30% nuclei were NF-kB positive, 20% nuclei were cleaved caspase 3 positive within the selected region of heart section). Whereas, in HM group maximum translocation of NF-kB and cleaved caspase 3 (66.67% nuclei were NF-kB positive**,** 58% nuclei were cleaved caspase 3 positive within the selected region of liver section and 60% nuclei were NF-kB positive, 48% nuclei were cleaved caspase 3 positive within the selected region of heart section) and the translocation was decreased in HM + EECGLH group (2.5% nuclei were NF-kB positive**,** 6% nuclei were cleaved caspase 3 positive within the selected region of liver section and 5% nuclei were NF-kB positive**,** 7.5% nuclei were cleaved caspase 3 positive among within the selected region of heart section in the fixed region (Fig. 6). Therefore, EECGLH supplementation in HM fed group decreased nuclear translocation of NF-kB (p65) and cleaved caspase 3 expressions. Thus, immunohistochemistry data further justified the finding of immunoblot data (Fig. 7).

### Effects of EECGLs on the expression of apoptosis related proteins and inflammation related genes

To further investigate the molecular mechanisms of the most effective dose of EECGL (EECGLH, revealed by the above statistically significant data level) on HM (the most significant observations were observed from the above data and statistical significant level) induced changes, in attenuating the progression of NAFLD, protein and mRNA expression levels of several apoptosis-related proteins and inflammation-related genes expressions were determined by Western blot analysis and RT-PCR (Fig. 7). mRNA expression of iNOS, TNF-α and protein expression of Bax, cleaved caspase 3, cleaved caspase 9, p21, p53 were significantly up regulated (*P* < 0.01) in the HM fed group with compare to the control group (NC) in both isolated hepatocytes and cardiomyocytes respectively. Furthermore, EECGLH supplementation significantly blunted the elevated mRNA and protein expressions (*P* < 0.01) with compare to the HM fed group. On the contrary, hepatic and cardiac mRNA expression of eNOS and protein expression of Bcl2, peroxisome proliferator-activated receptor-α (PPAR-α), peroxisome proliferator-activated receptor-γ (PPAR-γ) were significantly down regulated in the HM fed rats with compare to the NC group but the supplementation with EECGLH significantly up regulated those decreased expression of both mRNA and protein expression (*P* < 0.01) in contrast to the HM fed rats (Fig. 7A–G).

## Discussion

NAFLD mediated systemic malfunction has now become a global problem for common people. Several studies showed that individually both HLD and MSG has been associated with OS mediated systemic damage^1,6^. Although antioxidant play a crucial role in such pathophysiological state; but elevated production of ROS with altered balance of free radicals and endogenous antioxidants in the body resulted OS mediated systemic damage due to lack of endogenous antioxidants to combat against OS and OS mediated tissue damage. Likewise, upon treatment with HM caused generation of ROS and thereby OS mediated systemic damage which altered redox-status. Due to the major side effects of synthetic drugs the present study was design with plant based supplementation as an alternative strategy which further offers protective impacts against HM induced cellular damage. Therefore, attention was focused on EECGL as natural antioxidant to fight against the HM induced systemic anomaly.

Our investigation aimed to evaluate the effect of EECGL on HM induced NAFLD mediated anomalous situation and to explore the mechanism through which EECGL play an ameliorative role on HM induced systemic damage. Here, we used coconut oil, vanaspati ghee as the source of saturated fats and hydrogenated fats; MSG used as flavor enhancing reagent and in combination of these HLD and MSG creates a NAFLD model. We found that HM in combination with EECGL: (a) attenuated the increased lipogenesis, hyperlipidemia with disturbances in adiponectin, leptin level by HM; (b) up regulated the reduced expression of PPAR-α and PPAR-γ by HM; (c) attenuated the increased ROS generation by HM; (d) blunted the elevated levels of IL-6, IL-1β, TNF-α, TGF-β, hs-CRP with escalated the level of IL-10 and thereby inhibited inflammation with nuclear translocation of NF-kB (p65) in HM fed group; (e) attenuated the activated p53 and p21 with huge sub G0/G1 population by HM fed rats; (f) inhibited the activated the mitochondrial death pathway associated with loss of ΔΨm by HM fed rats, and thereby caspase-3 and 9 activation, increased Bax with decreased Bcl2 expression.

Our study mainly focused on the protective properties of EECGL against HM induced systemic damage in male rat model. HPTLC and GCMS results, revealed that EECGL have significant amount of β-carotene, (E)-γ-Atlantone, aR-Turmerone, Linolenic acid, β-Turmerone, Germacron, Ergost-5-en-3β-ol , δ-Tocopherol and Farnesol like known bioactive compounds with other unknown compounds. Accumulating evidences suggested that these phytocompounds have significant antioxidant, free radical scavenging^39–45^, anti-dyslipidemic^46,47^, hypoglycemic^48^, anti-inflammatory^39,41,49–53^, anti-apoptotic^45,52^, hepatoprotective^44,54^ and cardioprotective^39,47^ properties respectively. These compounds also acts as therapeutic agents for oxidative stress mediated systemic anomalies^49,52,54^ and other various health related disorders^49,55–57^. Earlier reports also proposed that *Coccinia grandis* have significant anti-diabetic, anti-dyslipidemic, anti-oxidative and hepatoprotective impacts^58,59^. Thus presence of carotenoids, phytosterols, sesquiterpenoids, acyclic sesquiterpene, vitamin, essential fatty acid and other active compounds in EECGL may be responsible for its potential ameliorative impacts on HM induced systemic damage via inhibition of OS, inflammation and apoptosis related pathways by regulating the redox-equilibrium. The synergistic impacts of such active compounds presence in EECGL offer as an alternative therapeutic strategy of management against HM induced systemic anomalies.

In present study, NAFLD model was set up by utilizing HLD and MSG which further stimulates NAFLD mediated with hepatic and cardiac damage due to the high content of saturated fatty acids (e.g., palmitic acid, lauric acid, caprylic acid, myristic acid, etc.), trans fatty acids and hydrogenated fats in HLD^1^ in HM combination. Result uncovered that disturbances in LDL and HDL level in HM group experienced hyperlipidemia related with lipid metabolic aggravations, finally increased lipogenesis and decreased lipolysis. Banerjee A et al. suggested that higher TC, TG, LDL, VLDL and reduced HDL were good indicator of cardiovascular damage^1^. Similar type of results was also observed in the current investigation. Moreover, increased TC and TG caused lipid accumulation in the hepatic and cardiac tissue to stimulate systemic damage which was also well corroborated with earlier study^1^. On the contrary, EECGL supplementation offered anti-dyslipidemic impacts on HM induced dyslipidemia to combat against hepatocellular and cardiovascular damage which was well corroborated with previously mentioned anti-dyslipidemic and hepatoprotective activity of *Coccinia grandis*^58,59^. Furthermore, histopathological observations on both H & E staining and Oil red O staining demonstrated serious hepatic steatosis and cardiac tissue damage with lipid droplets in HM groups contrasted to the control group due to higher lipid profile and increased cellular toxicity marker such as ALT, AST, ALP, LDH and CK-MB level. Earlier report revealed that the serum AST and ALT levels are gentle to tolerably rise in patients with NAFLD^60^. The ALT, AST and ALP levels were escalated by 72.51%, 68.01% and 40.98% respectively, in the HM fed group with the NC group, demonstrated fruitful foundation of the NAFLD model. Moreover, increased ALT, AST, ALP, LDH and CK-MB also indicated myocardial infarction (MI)^1^; similar type of effects were also observed in HM treated animals. Here also EECGL restored the increased cellular toxicity marker enzymes, improve the tissue architecture of liver and heart, and reduced the occurrence of MI to offer as systemic protective supplement against HM induced systemic damage.

Insulin resistance is a typical feature in NAFLD patients^61^ which was also evident in the present experiment. Furthermore, OS and cytokines played a silent key role in mediating the progression of NAFLD from steatosis to nonalcoholic steatohepatitis (NASH), fibrosis and cirrhosis which further prompts cardiovascular ill effects or coronary heart disease. In NAFLD, ROS is generated by fatty acid oxidation^61^. In the in vivo experiment, HM stimulates immense generation of ROS linked with cellular lipid and DNA molecules to cause lipid peroxidation and single strand to double strand break in DNA respectively. ROS attack polyunsaturated fatty acids and start lipid peroxidation and NO inside cells, resulting in the formation of aldehyde results, for example, MDA. Aldehyde by-products like MDA, is formed by lipid peroxidation within cells when ROS attacked polyunsaturated fatty acids. These particles can possibly diffuse from their destinations of cause to achieve far off intracellular and extracellular targets, and thus intensifying the impacts of oxidative stress^62^. Moreover, pro-inflammatory responses have been linked with OS and several studies suggested about the connection between OS and systemic damage^1,6^. In the present study, HM induced OS and thereby lipid peroxidation as indicated by increased TBARS level in both hepatic and cardiac tissue homogenate as well as cellular level. Generation of ROS disturbed the normal homeostasis by altered the balance between endogenous antioxidant and prooxidant, which ultimately leads to OS. The results of our study demonstrated a marked reduction of HM induced OS with EECGL supplementation, suggesting the role of EECGL in preventing HM induced systemic anomalies either by directly acting as a ROS scavenger or by indirectly modifying the cell signalling system which altered the redox-equilibrium. The present investigation revealed that the degree of OS generated by HM and the endogenous antioxidant to counteract this effect in HM induced OS in liver and heart. Furthermore, the present study also evaluated the underlying cause of OS in liver and heart with respect five key markers including TBARS, NO, SOD, CAT and GSH which provided the information about the efficacy of EECGL to combat against the HM induced degenerative impacts. Because HM induced the alteration of cellular functions via depletion of redox-homeostasis; however detailed investigation of hepatic and cardiovascular system with respect to intracellular antioxidants with their corresponding activities were important to assessed the effectiveness of EECGLs on experimental animals. HM has been associated with a significant reduction of endogenous enzymatic and non-enzymatic antioxidants level (i.e., SOD, CAT and GSH) and increased reactive free radicals and electrophiles, which ultimately results in OS mediated systemic dysfunctions. Reduced levels of endogenous antioxidant promoted OS and thereby OS-mediated damage of macromolecules in the hepatic and cardiac tissue. However, the administration of EECGLs markedly escalated the endogenous antioxidants SOD, CAT and GSH in the liver and heart of HM treated rats approximately in the normalcy. EECGL also blunted the HM induced generation of free radicals and lipid peroxidation in the liver and heart, indicated its direct connection with antioxidant and free radical scavenger in vivo due to the anti-oxidative property and presence of various antioxidant compounds in the EECGL via regulating the redox-homeostasis. This anti-oxidative activity of *Coccinia grandis* was well corroborated with earlier study^59^. The antioxidant capability of EECGLs can be suggested as protective supplement to improve liver functional status in NAFLD rats and cardio-toxicity marker enzymes to reduce cardiac damage. The harmony between cytokines/adipocytokines and anti- and pro-inflammatory activities play an important role in the progression of NAFLD mediated systemic disorders. The pro-inflammatory cytokines TNF-α and IL-6 were fundamentally associated with the different parts of pathophysiology of human NAFLD, and were the significantly escalated hepatic generation of hs-CRP, fibrinogens and other intense stage proteins. The increased level of hs-CRP was a good indicator of cardiovascular disorder in NAFLD, especially in those with NASH patients^63^. The powerful TNF-α neutralizing and anti-inflammatory adipocytokine is Adiponectin. In vitro and experimental animal studies have shown the significance of this mediator in impeding IR and inflammation. The anti-inflammatory impacts of adiponectin was interceded by the concealment of TNF-α synthesis and the induction of anti-inflammatory cytokines IL-10 receptor foes. Zaitone et al.^64^ have also shown that boswellic acids and pioglitazone improve insulin affectability and blunted the liver index, activity of liver enzyme action, and the serum TNF-α and IL-6 levels in rats treated with regular consumption of high lipid diet. Moreover, Makabe et al.^51^ suggested anti-inflammatory potential of sesquiterpenes from *Curcuma zedoaria* where germacrone was an active compound; similarly with the previous information EECGLs fundamentally reduced the IL-6, IL-1β, TGF-β, TNF-α and hs-CRP levels and decreased the leptin with increased adiponectin and IL-10 level, proposed anti-inflammatory potential of EECGL against HM induced inflammatory response, where germacrone also present as an active compound in EECGL.

Furthermore, inflammation and discharge of collagen cause damage of hepatocytes that ultimately leads to the development of NASH from steatosis. In case of liver cirrhosis 1/5th of the patients with NASH, hepatocytes are replaced by collagen tissue^65,66^. TGF-β plays a crucial role in the progression of fibrosis of liver^61^. Song et al*.*^61^ demonstrated that up regulation of the pro-fibrogenic TGF-β is associated with acute necro-inflammation and fibrosis in a rat model of steatohepatitis similar type of effect was also observed in the present study. Moreover, decreased level of TGF-β was found in EECGL supplementary group in dose dependant manner which blunted the progression of inflammation and development of fibrosis in the present investigation. Therefore it can be suggested as potential ameliorative supplement against NAFLD mediated systemic damage. Moreover, hepatic and cardiac tissue injury were associated with increased level of IL-6 and TNF-α which were linked with the generation of ROS induced by HM. Increased level of IL-6 and TNF-α promoted tissue damage through inflammatory pathway via this vicious cycle. In the present investigation, HM induced OS caused activation of intracellular transcription factor like NF-kB (p65), which was responsible for inflammation^67^. Expression level of different pro-inflammatory mediators like TNF-α, IL-1β and IL-6 were increased on binding of specific sequence of promoter region of the target gene with NF-kB^68,69^; hence altered pro-inflammatory/anti-inflammatory factors further activated intracellular NF-kB (p65) in HM fed groups to promote inflammation. On the contrary, EECGL beneficially blunted the pro-inflammatory factors with boosted anti-inflammatory factors to attenuate HM induced inflammation via inhibition of NF-kB (p65) signaling pathway. Therefore, EECGL offered as an anti-inflammatory supplement which fights against HM induced inflammation.

We also observed that in case of HM fed animals sub G0/G1 population was significantly (hepatocytes: *P* < 0.001, cardiomyocytes: *P* < 0.01) increased as compared to the NC group. HM induced apoptosis by triggering increased sub G0/G1 phase of cell cycle and finally arrest of G0/G1 phase; Banerjee A et al. already showed that HLD induced necro-apoptosis by increased sub G0/G1 population^1^. Therefore, the present study revealed that HLD in combination with MSG provoked apoptosis by increased sub G0/G1 population in cell cycle phase, which was well supported with caspase 3 and 9 activation with altered Bax/Bcl2 level.

In addition we have also checked hepatic and cardiac mRNA expression of iNOS, eNOS and TNF-α (involved in inflammations) and levels of protein expressions of PPAR-α, PPAR-γ, p21, p53, Bcl2, Bax, cleaved caspase 3, cleaved caspase 9 (associated with dyslipidemia, metabolic disturbances, oxidative stress and apoptosis) in this present experimental set up; increased mRNA expression of iNOS, TNF-α were significantly (*P* < 0.01) down regulated and decreased mRNA expression of eNOS was significantly (*P* < 0.01) up regulated by EECGLH in both isolated hepatocytes and cardiomyocytes. Furthermore, EECGLH also augmented the reduced protein expression of Bcl2, PPAR-α, PPAR-γ and down regulated the elevated expression of p21, p53, NF-kB (p65), Bcl2, Bax, cleaved caspase 3, cleaved caspase 9 in HM fed rats in both isolated hepatocytes and cardiomyocytes as compared to the HM group (*P* < 0.01).

The rate of cell division is important for normal growth and development of cells. p53 is considered as one of the checkpoint regulator for the G1 and G2 phases. Furthermore, it actuates programmed cell death by halting G0/G1 phase after damage of DNA. Phosphorylation of p53 boosted DNA damage. Phosphorylation of p53 causes DNA damage via p-DNAPKcs and p-ATM kinases^70^. The present study demonstrated HM caused increased cell population in sub G0/G1 phase of cell cycle, reduction of MMP, augmented the level of pro-inflammatory factors with reduced anti-inflammatory factors, up regulation of apoptotic mediators like p53, p21, cleaved caspase 3, cleaved caspase 9 and Bax simultaneously with down regulation of anti apoptotic Bcl2 which further leads to programmed cell death via mitochondrial caspase mediated apoptotic pathway. On the other hand, regulation of survival, proliferation and growth of cell including anti-apoptotic family like Bcl2 are regulated by AKT/PI3K^71^. Survival of cells and inhibition of p53 is associated with the phosphorylation of AKT^72^. p53 and AKT exerts their important role by showing their opposite effects on programmed cell death. Considering the above facts, we hypothesized that p53-AKT duality play a key role in controlling survival of cell or death.

Subsequently all these events are channeled to the enhanced expression of p21 and p53 which are thought to be pivotal cellular response elements to DNA damage^73^. p53 and p21 expression were escalated in anticipation to lasting DNA damage leading to cell cycle arrest, which further ensures damaged DNA repairing^74^. In the present experimental set up, compared to control animals expression of pro-apoptotic Bax was enhanced in HM fed rats simultaneously with reduced expression of anti apoptotic Bcl2. Thus we speculate that combined high lipid diet and mono sodium glutamate treatment interfere with the progression of cell cycle possibly targeting the p53/p21 signaling and cause apoptosis by inducing disequilibrium in the Bcl2/Bax ratio. This speculation was further underlined by higher number of cells in sub G0/G1 population in HM fed animals as subG0/G1 cell population was more likely to undergo apoptosis^1^. Earlier reports reveled that p53 plays a crucial role in regulating G1 and G2 phases of the cell cycle^75^ and mediates transcriptional activation of Bax^76,77^. Similar observations were also evidenced in this study. Thus p53 mediated enhanced Bax expression form pores in the membrane of mitochondria leading to leakage of cytochrome C. This cytochrome C in succession activates the intrinsic pathway of apoptosis as demonstrated by enhanced level of active caspase 3 in HM fed animals compared to control animals. Here, EECGL possessed an important role to reduce HM induced apoptosis via inhibition of mitochondria mediated apoptotic pathway by regulating the different phases of cell cycle and Bax/Bcl2 ratio, restored MMP, blunted the elevated expression of p53, p21, cleaved caspase 3 and caspase 9. Therefore, it can be suggested that EECGL have significant anti-apoptotic potential against HM induced programmed cell death. Moreover, EECGLH attenuated the development of hepatic steatosis and progression to fibrosis and thereby limits NASH mediated systemic damage.

Additionally, transcription factors PPARs are thought to modulate the expression of gene associated with lipid digestion, inflammation and energy homeostasis. Experimental proof demonstrated PPAR-α as the prime controller of hepatic β-oxidation and microsomal Ω-oxidation which were markedly decreased by high lipid diet. Earlier report suggested that phytosterol ester activated PPAR-α and PPAR-γ to treat NAFLD^61^ which was also well corroborated with the present study by using EECGL where phytosterol also present as an active compound to combat against HM induced NAFLD mediated systemic damage. Furthermore, advantageous impacts of EECGL to restored adiponectin level and reduced IR via activation of PPAR-α and PPAR-γ. The consequences of our study demonstrated that medications with the most effective dose of EECGL, i.e., EECGLH actuated PPAR-α and PPAR-γ expression. Considering the previously mentioned role of PPAR-α and PPAR-γ, they may be the target proteins that are increased by EECGLH on protection against the development of NAFLD mediated systemic damage. In outline, treatment with EECGLs improved liver and heart histology, diminished hepatic and cardiac total lipids, reduced the serum ALT, AST and ALP levels. EECGLs adequately blunted the event and development of NAFLD by bringing down the LDL level and raised the HDL level, improving OS and regulating key cytokines. These advantages might be related with modifications in the hepatic and cardiac mRNA of certain inflammatory genes and certain inflammation, metabolism, and apoptosis related marker proteins. Further human experiments are required to decide the capability of EECGLs or more explicitly, the most effective dose of EECGL, i.e., EECGLH as helpful supplement for the counteractive action and development of NAFLD mediated CVD, OS and apoptosis.

**Figure 8:**
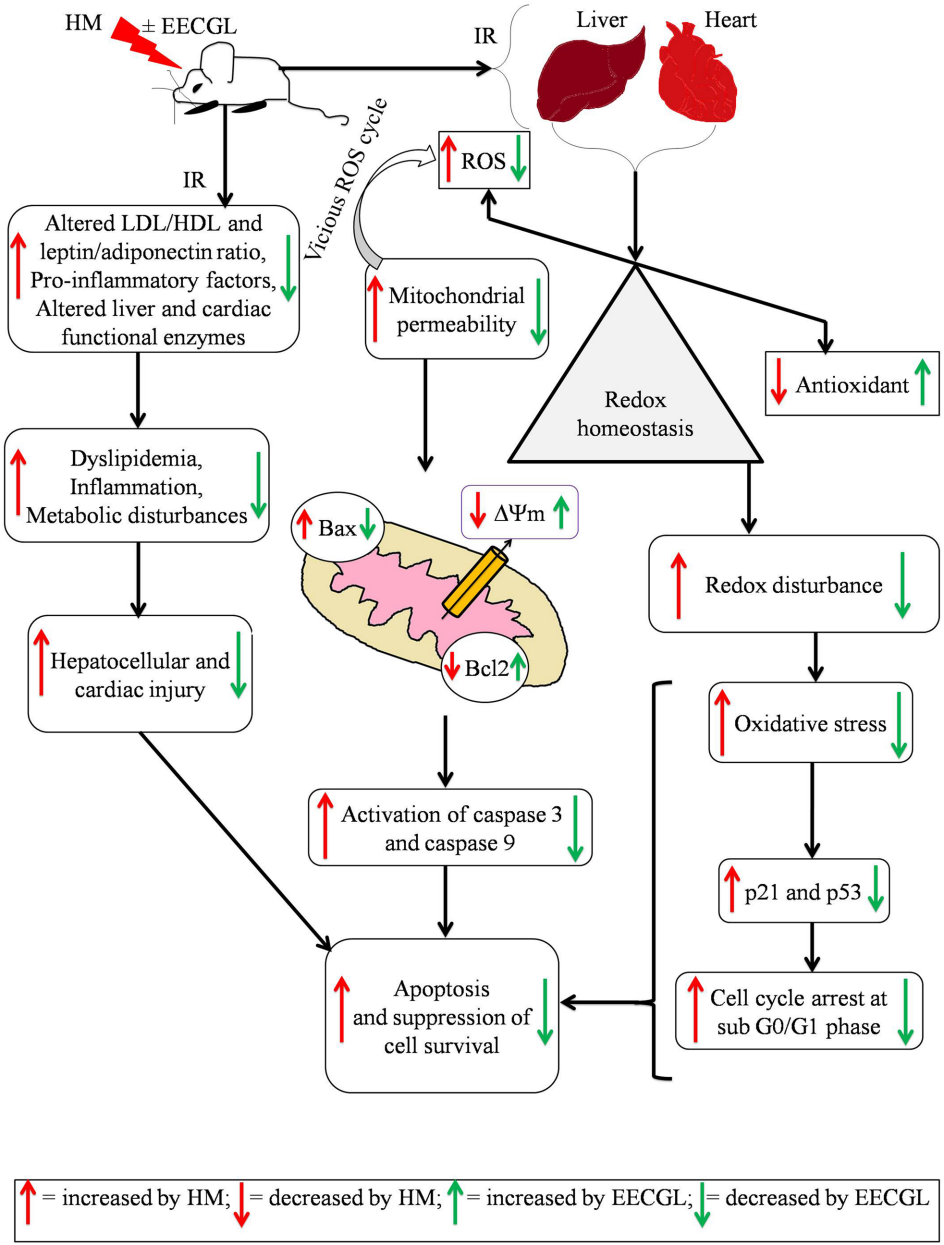
Schematic diagram of the hypothesis of the target pathway by which EECGL play preventive roles against HM induced NAFLD mediated systemic damage. The diagram represented the hypothetical pathways involved in high lipid diet along with monosodium glutamate induced liver, heart damage and a possible ameliorative efficacy of ethanol extract of *Coccinia grandis* leaves with its active compounds.

A therapeutic plant plays a viable strategy of management due to minimal side effects as compared to synthetic drugs, cheap in cost to treat NAFLD mediated systemic damage. Our study proposed that, HM induced systemic damage potentially ameliorated by EECGL via co-ordinated modulation of redox-guided signalling by suppression of NF-kB associated inflammation and mitochondrial caspase mediated programmed cell death. Moreover, it can be suggested that ethanol extract of *Coccinia grandis* leaves has enormous source of natural antioxidants and free radical scavengers; here EECGL acts as a potent natural supplement of antioxidants having in vivo ROS scavenging activity, with anti-dyslipidemic, anti-inflammatory, anti-apoptotic and also in defensive properties against consolidated impact of MSG mixed HLD induced NAFLD-mediated systemic damage (as shown in the Fig. 8). As enormous number of bioactive compounds have found in *Coccnia grandis*, it can be studied further as natural food constituents to avoid HM induced systemic anomalies. Furthermore, we are trying to isolate these phytocompounds from the leaves of *Coccinia grandis* and find out possible mechanism of action in animal model as well as human trial for preparing the novel drug from the EECGL. It would be a better ameliorative drug for the patient experiencing different disorders particularly HM linked NAFLD, hepatic steatosis and coronary heart disease. For the development of better, cheaper and alternative source of potent drug against NAFLD-mediated systemic damage, further studies are required.

## Supplementary information

Supplementary Information.
