# Supplementary material for: Mechanistic study of attenuation of monosodium glutamate mixed high lipid diet induced systemic damage in rats by Coccinia grandis
Source: Sci Rep. 2020 Sep 22;10:15443. doi: 10.1038/s41598-020-72076-6 (PMC7508805; doi:10.1038/s41598-020-72076-6)
Supplement: Supplementary file 1 — Supplementary Information. [file 41598_2020_72076_MOESM1_ESM.pdf]

Mechanistic study of attenuation of monosodium glutamate mixed high lipid diet induced systemic damage in rats by *Coccinia grandis*

Arnab Banerjee[1], Debasmita Das[1], Rajarshi Paul[1], Sandipan Roy[1], Ujjal Das[2], Samrat Saha[2], Sanjit Dey[2], Arghya Adhikary[3], Sandip Mukherjee[1], Bithin Kumar Maji[1*]

[1]Department of Physiology (UG & PG), Serampore College, 9 William Carey Road, Serampore, Hooghly-712201, West Bengal, India
[2]Department of Physiology, University College of Science, Technology and Agriculture, University of Calcutta, 92 Acharya Prafulla Chandra Road, Kolkata-700009, West Bengal, India
[3]Centre for Research in Nanoscience and Nanotechnology, Acharya Prafulla Chandra Roy Sikhsha Prangan, University of Calcutta, JD-2, Sector-III, Saltlake City, Kolkata-700098, West Bengal, India

[*]Corresponding author: Dr Bithin Kumar Maji
Designation: Associate Professor
Address: Department of Physiology (UG & PG), Serampore College, 9 William Carey Road, Serampore, Hooghly-712201, West Bengal, India
E-mail id: bm_scp@yahoo.in; Mobile: +91- 9433509890

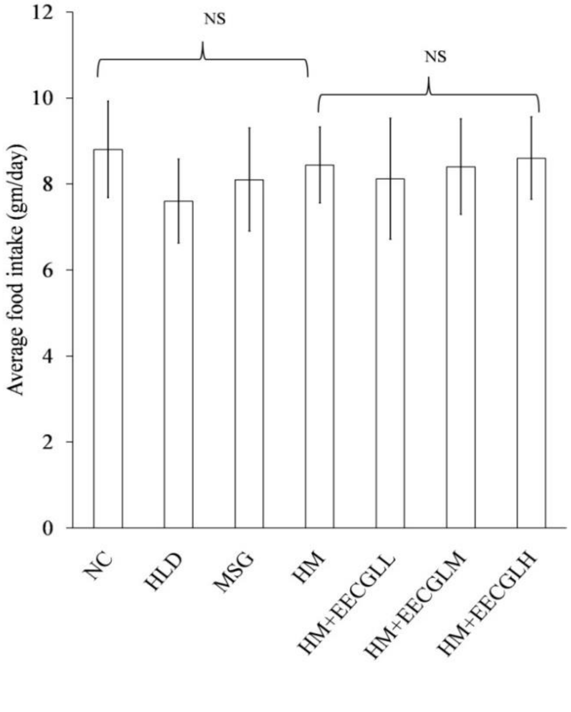

Supplemental Fig. S1: Average food intake among different groups of animals including NC, HLD, MSG, HM and EECGLs. Statistical comparison by Kruskal-Wallis nonparametric ANOVA test [not significant: NS]. Significance level based on Mann-Whitney U multiple comparison test: NC vs. HLD/MSG/HM: NS; HM vs. HM+EECGLL/EECGLM/EECGLH: NS.

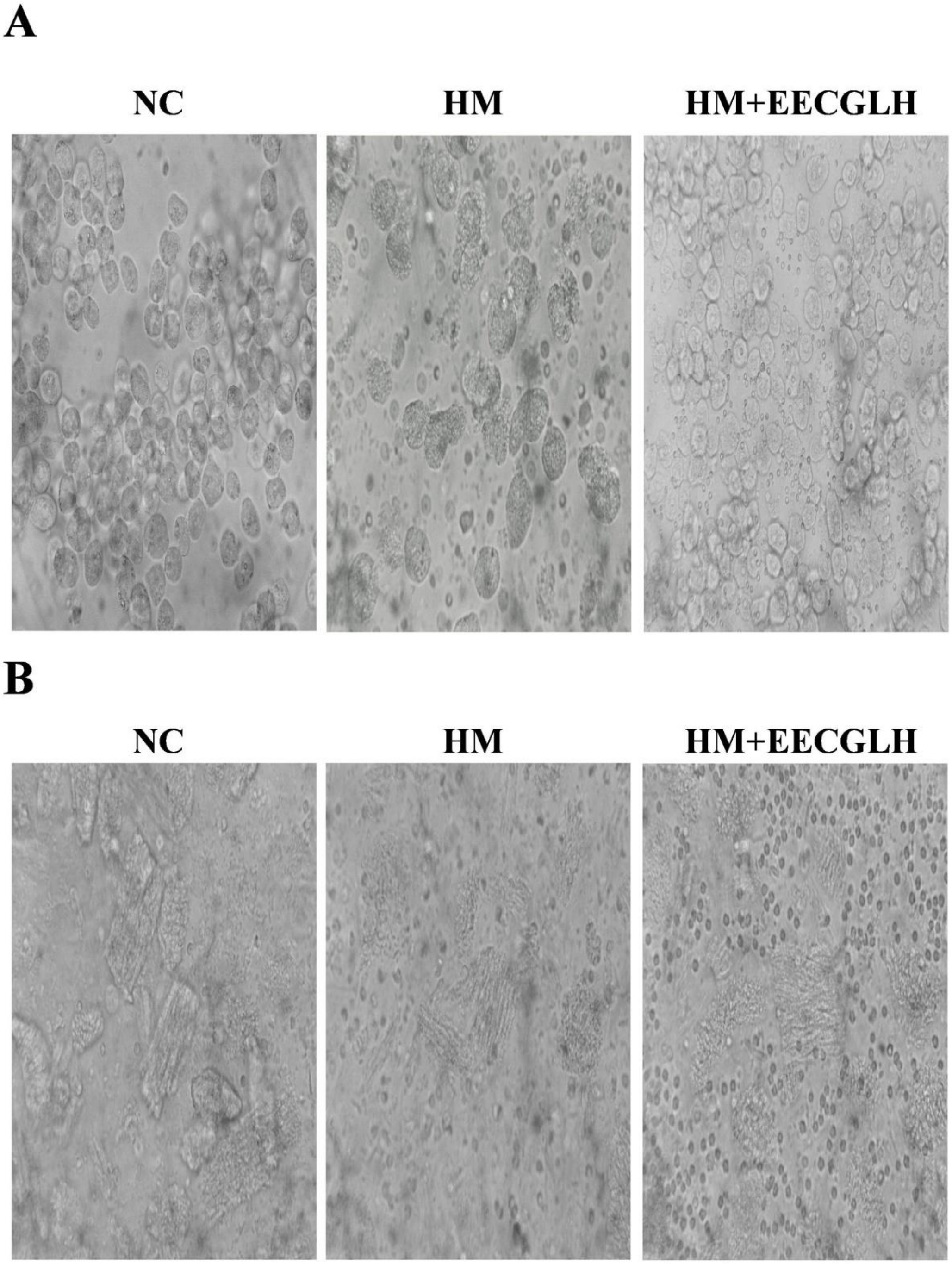

Supplemental Fig. S2: Isolated hepatocytes (A) and cardiomyocytes (B) of NC, HM and HM+EECGLH group.

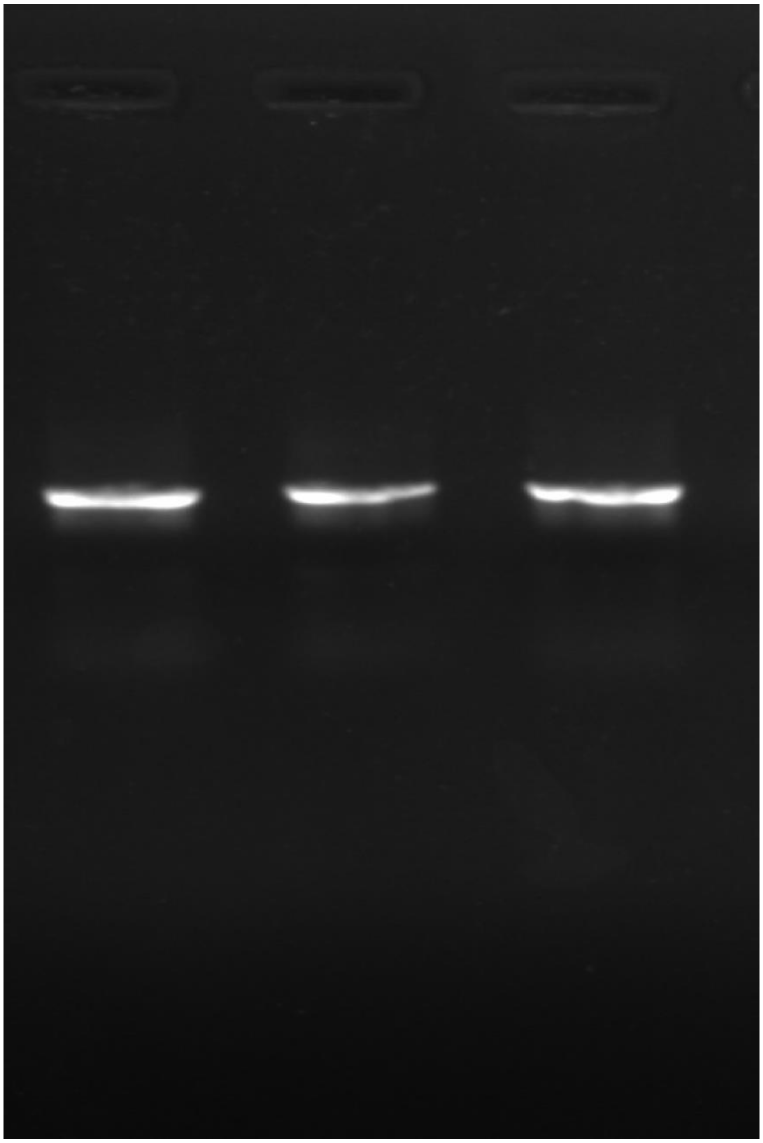

Supplemental Fig. S3: Full length photograph of 1.8% agarose gel with eNOS expression as PCR product of isolated hepatocytes after electrophoresis.

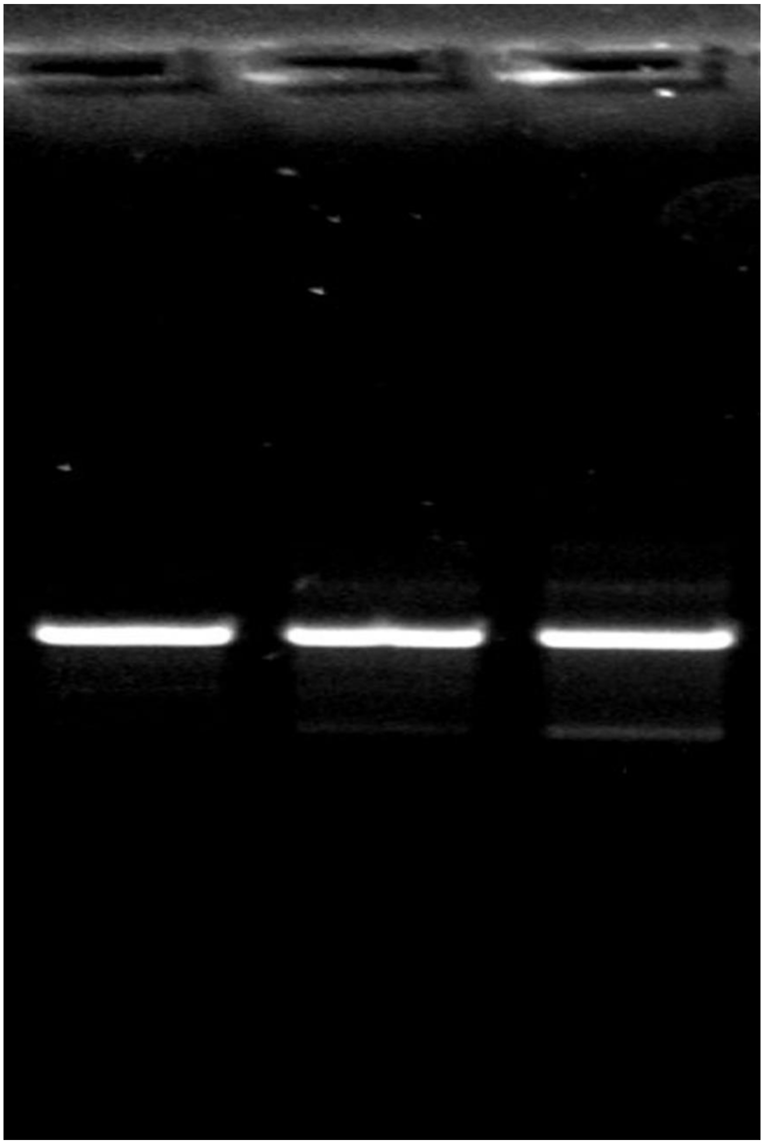

Supplemental Fig. S4: Full length photograph of 1% agarose gel with iNOS expression as PCR product of isolated hepatocytes after electrophoresis.

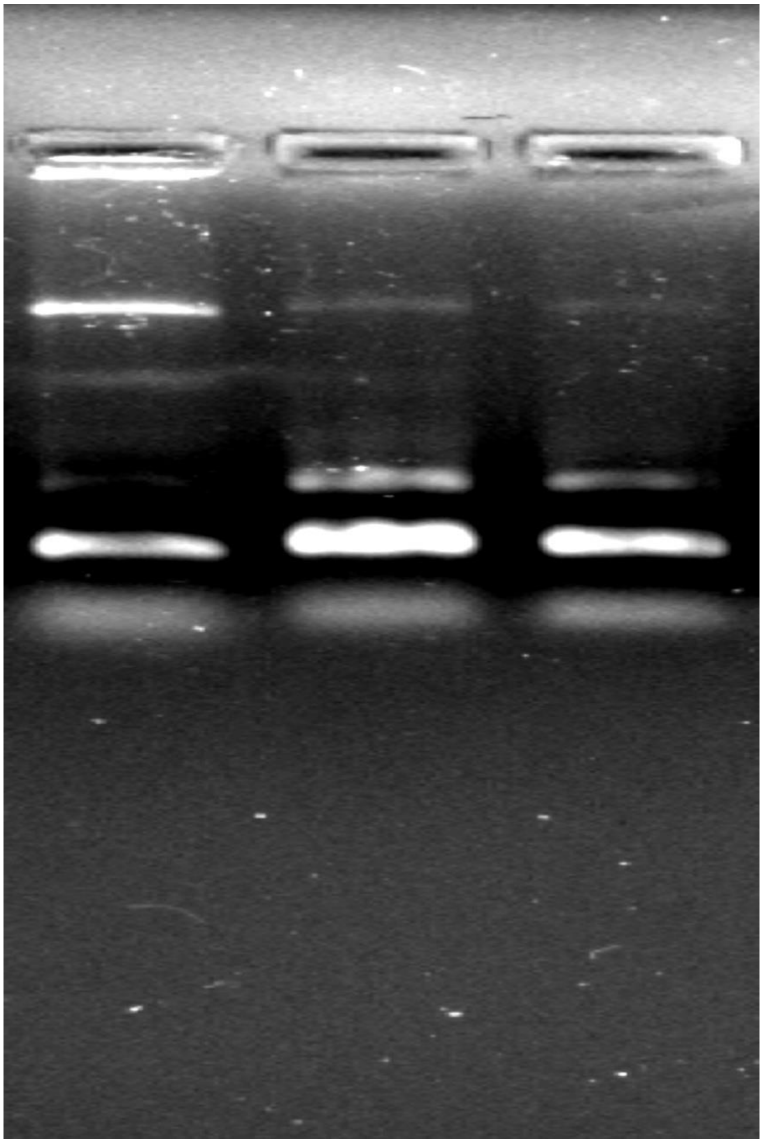

Supplemental Fig. S5: Full length photograph of 1.8% agarose gel with TNF-α expression as PCR product of isolated hepatocytes after electrophoresis.

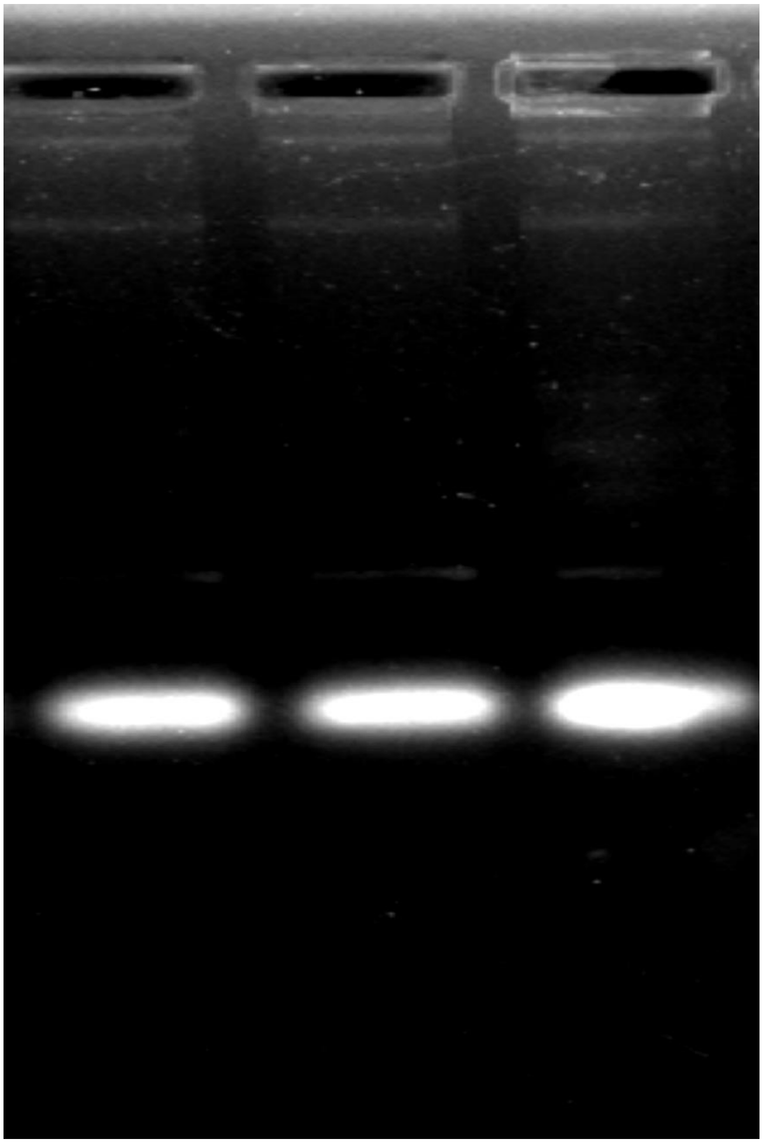

Supplemental Fig. S6: Full length photograph of 1.8% agarose gel with β-actin expression as PCR product of isolated hepatocytes after electrophoresis.

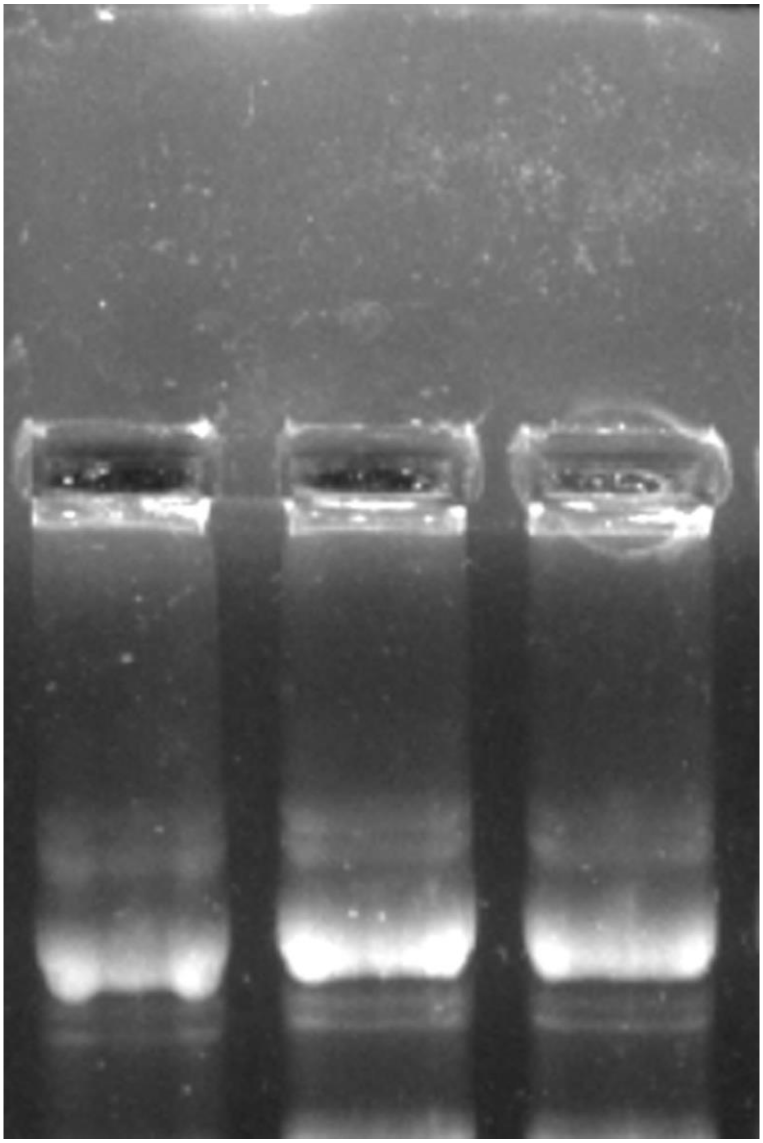

Supplemental Fig. S7: Full length photograph of 1.8% agarose gel with TNF-α expression as PCR product of isolated cardiomyocytes after electrophoresis.

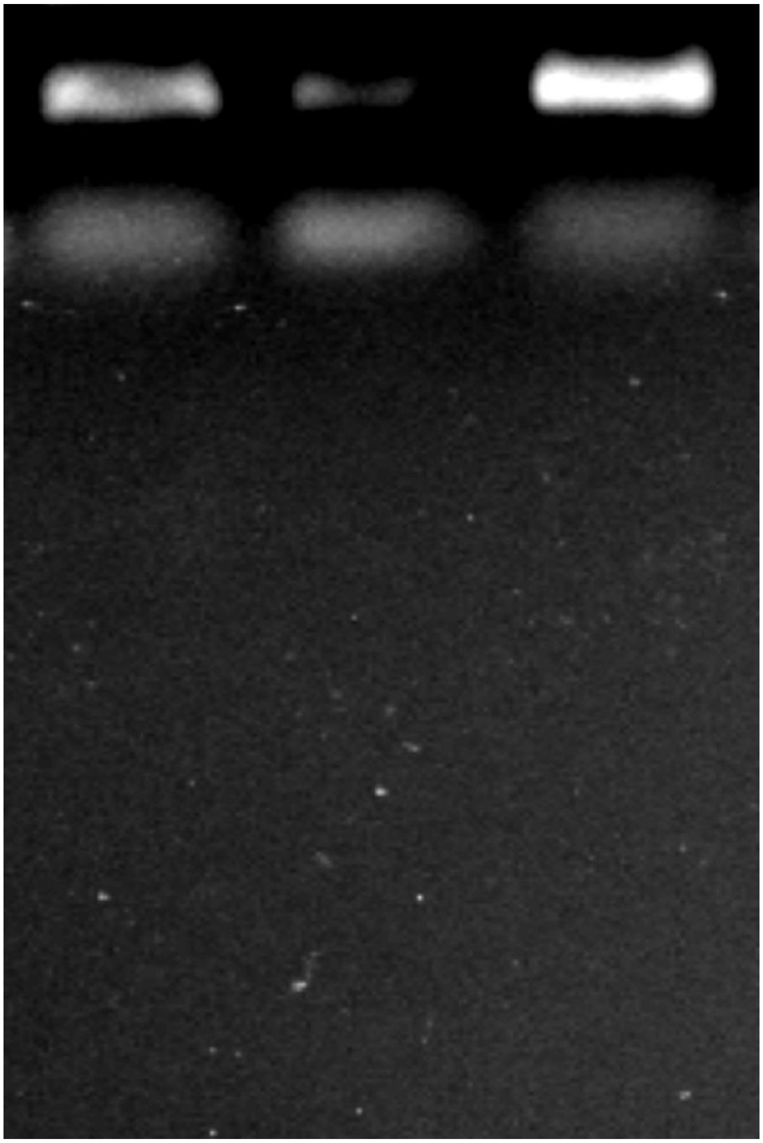

Supplemental Fig. S8: Full length photograph of 1.8% agarose gel with eNOS expression as PCR product of isolated cardiomyocytes after electrophoresis.

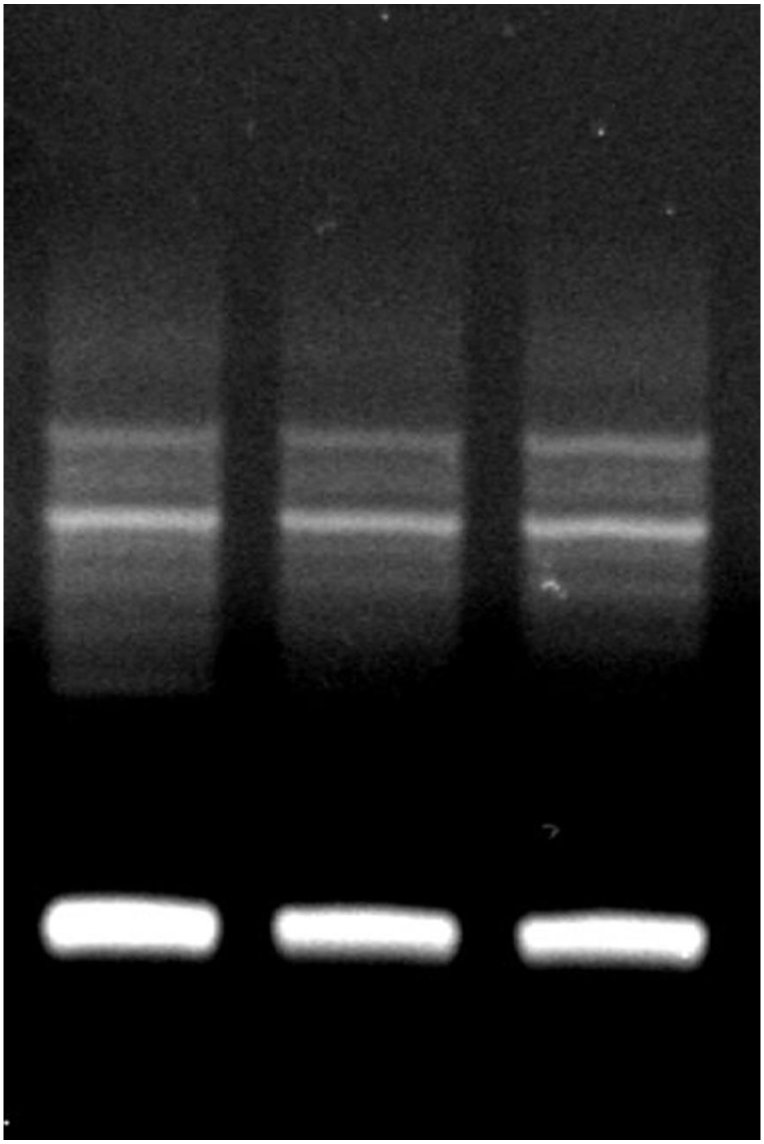

Supplemental Fig. S9: Full length photograph of 1.8% agarose gel with iNOS expression as PCR product of isolated cardiomyocytes after electrophoresis.

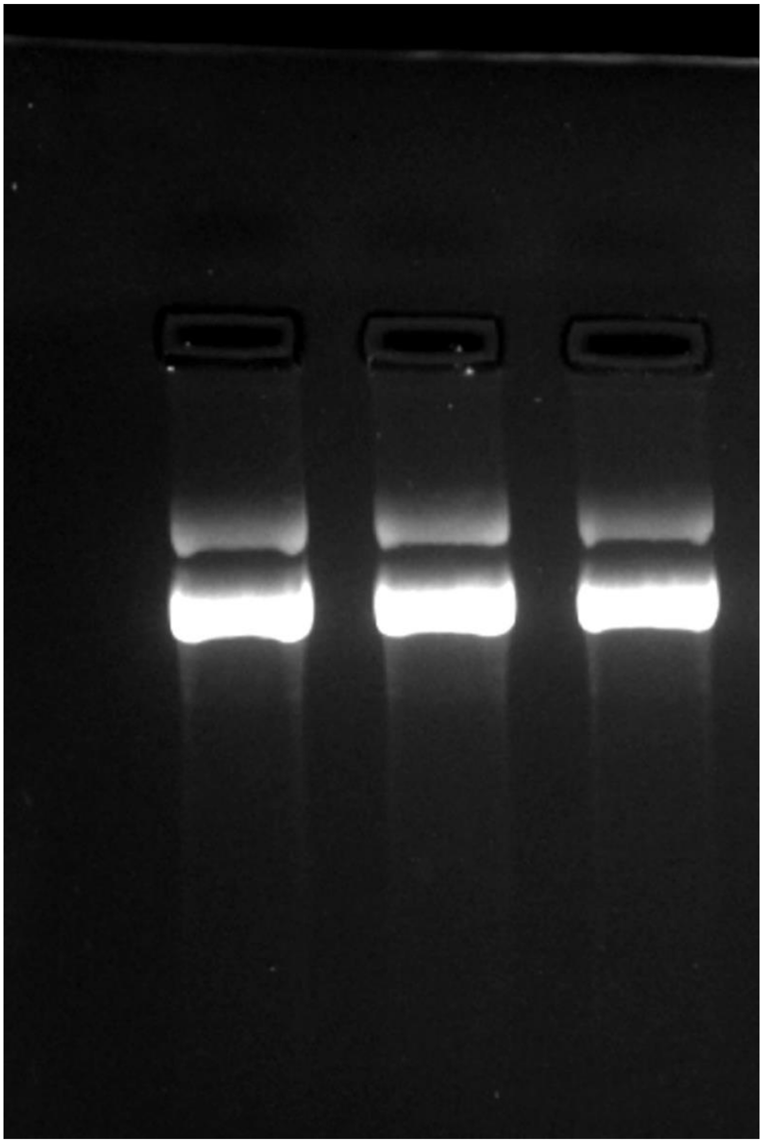

Supplemental Fig. S10: Full length photograph of 1.8% agarose gel with β-actin expression as PCR product of isolated cardiomyocytes after electrophoresis.

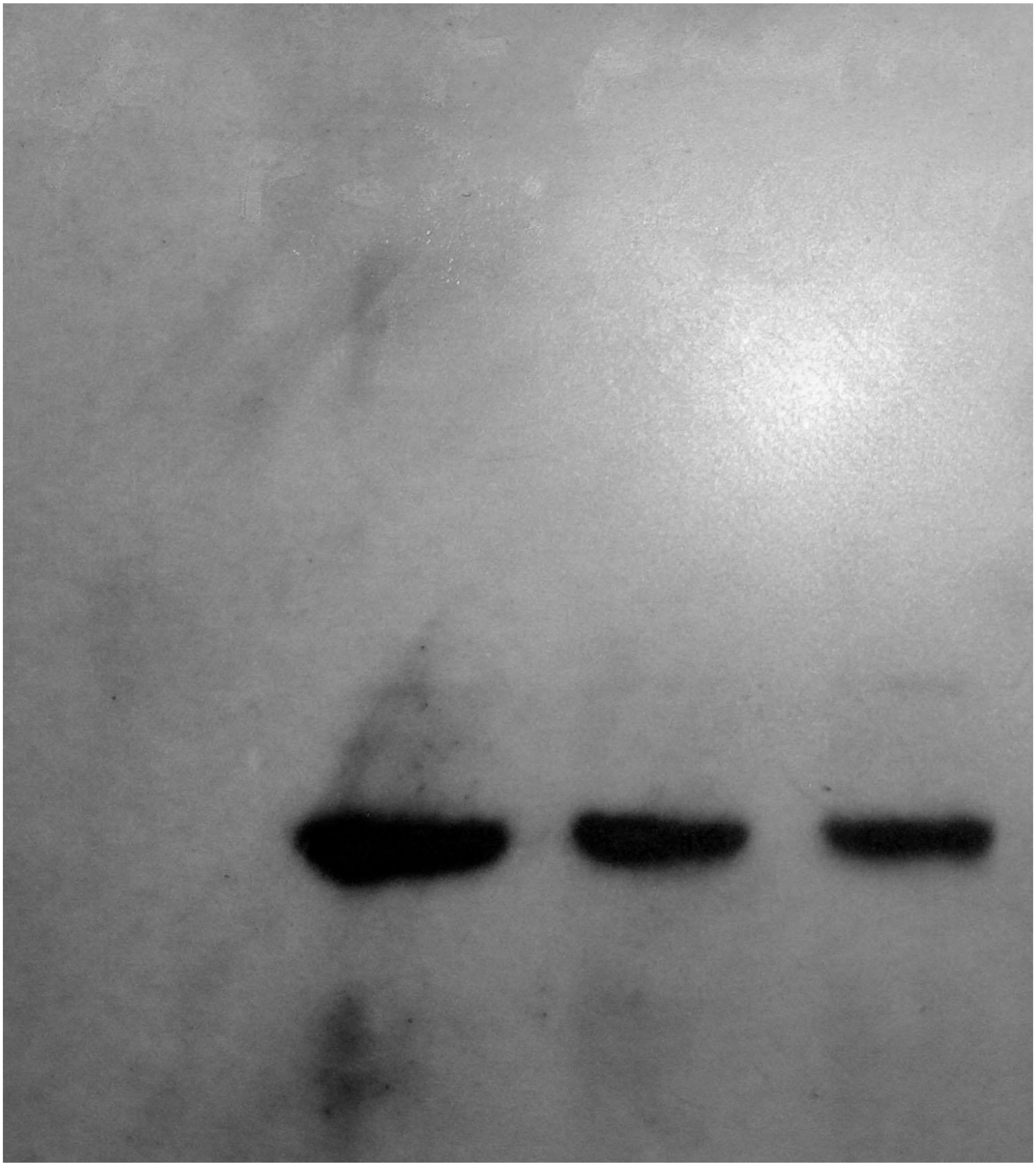

Supplemental Fig. S11: Full length photograph of western blot image with Bcl2 expression of isolated hepatocytes.

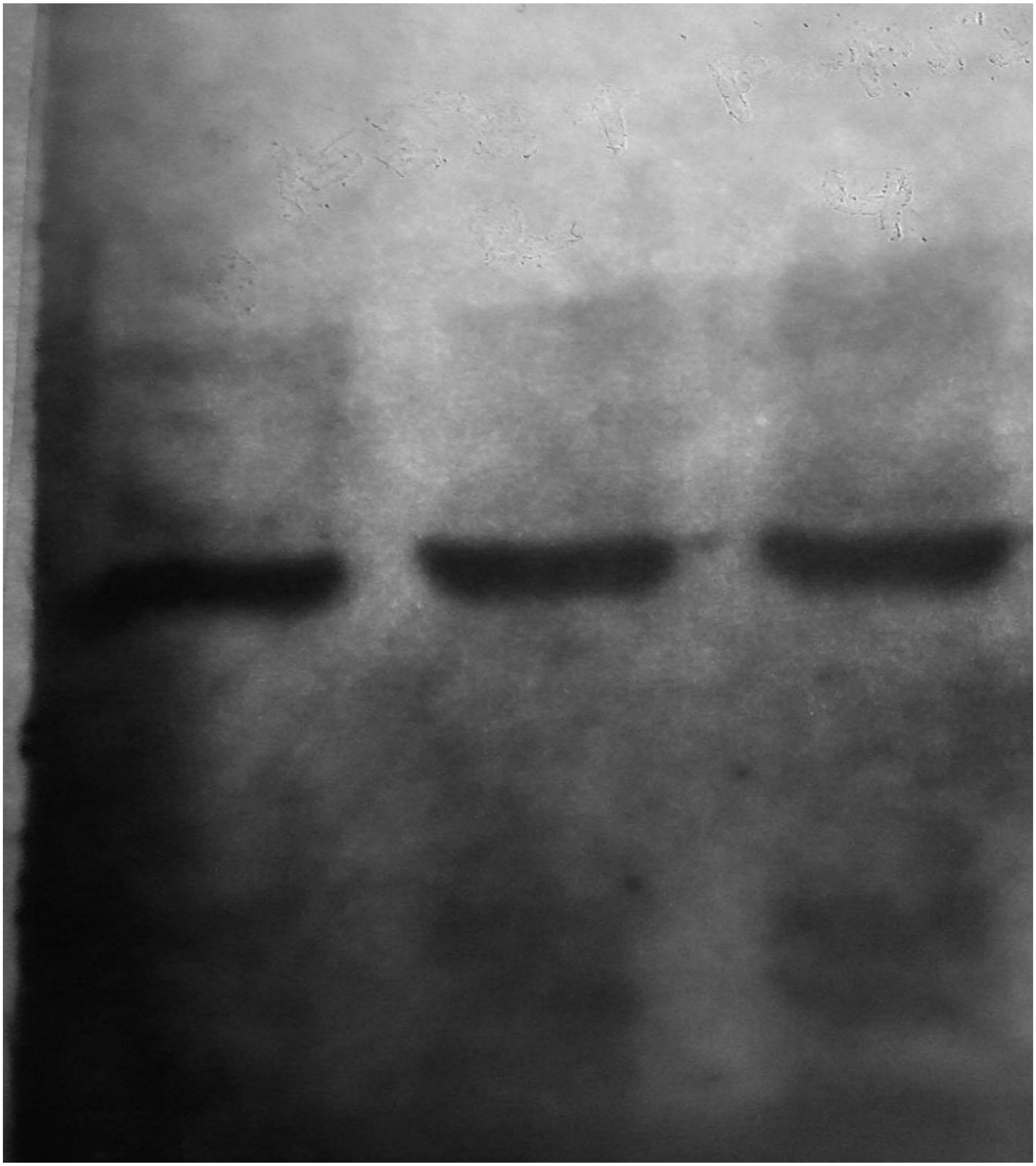

Supplemental Fig. S12: Full length photograph of western blot image with Cleaved caspase 9 expression of isolated hepatocytes.

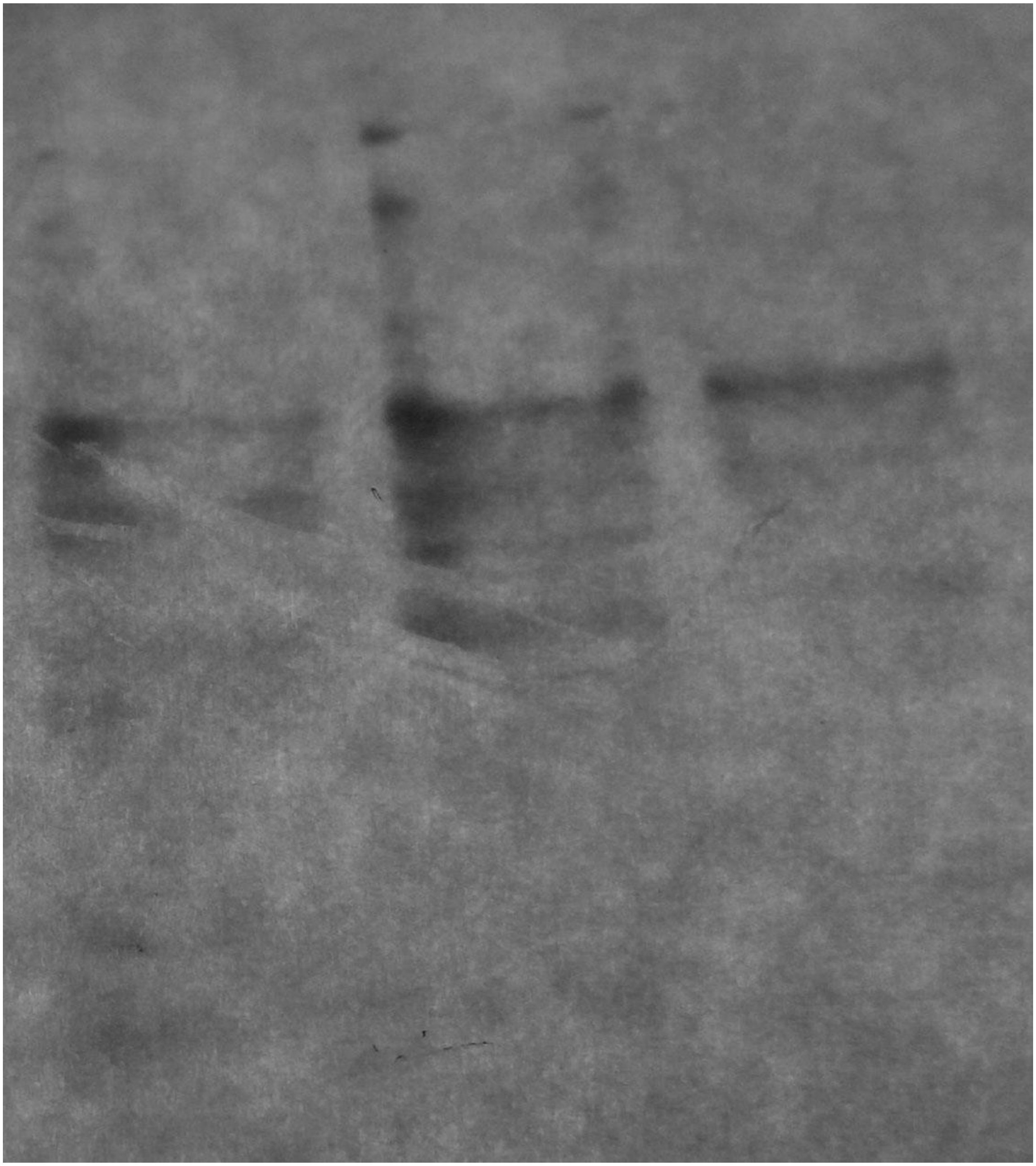

Supplemental Fig. S13: Full length photograph of western blot image with p21 expression of isolated hepatocytes.

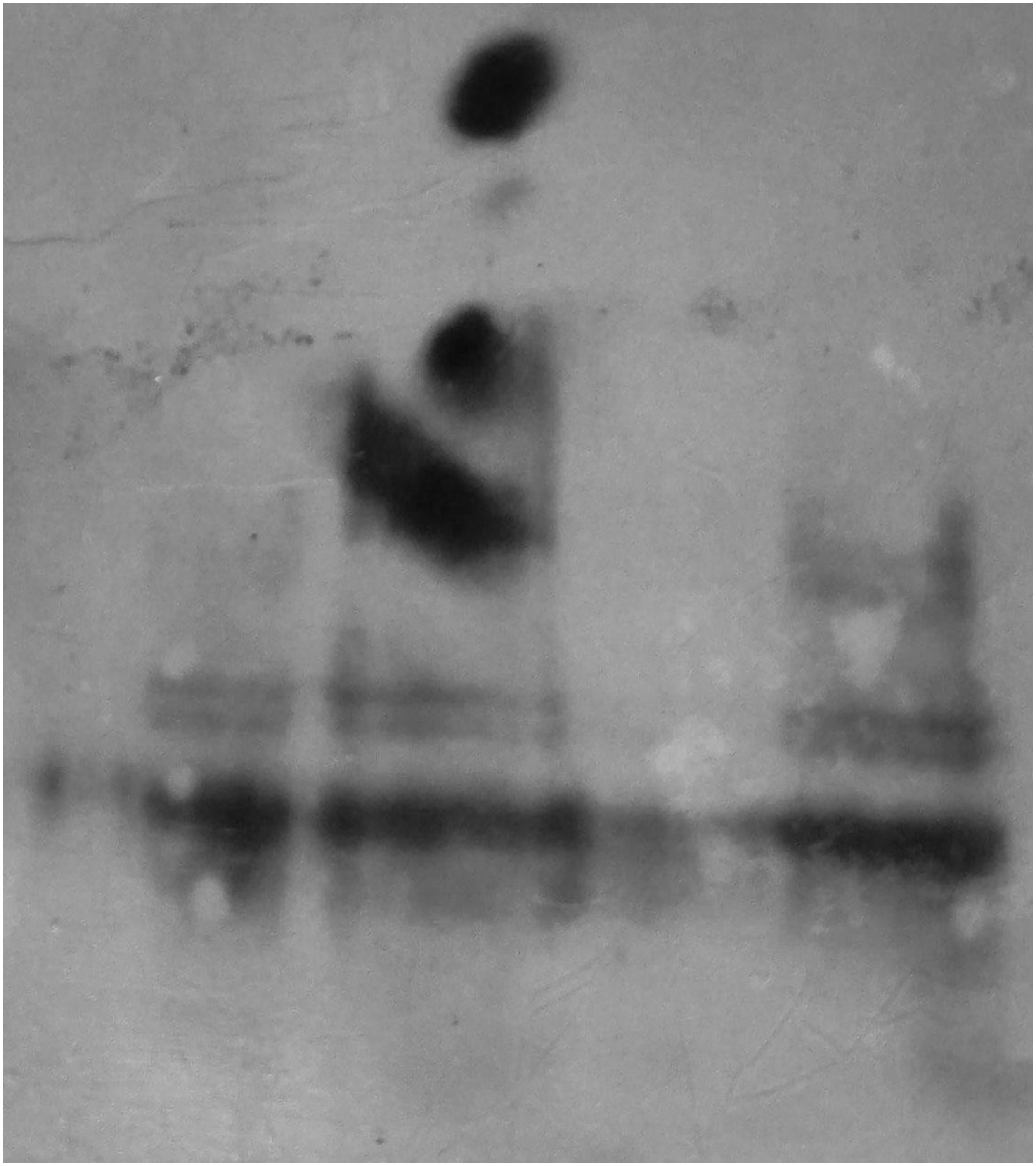

Supplemental Fig. S14: Full length photograph of western blot image with p53 expression of isolated hepatocytes.

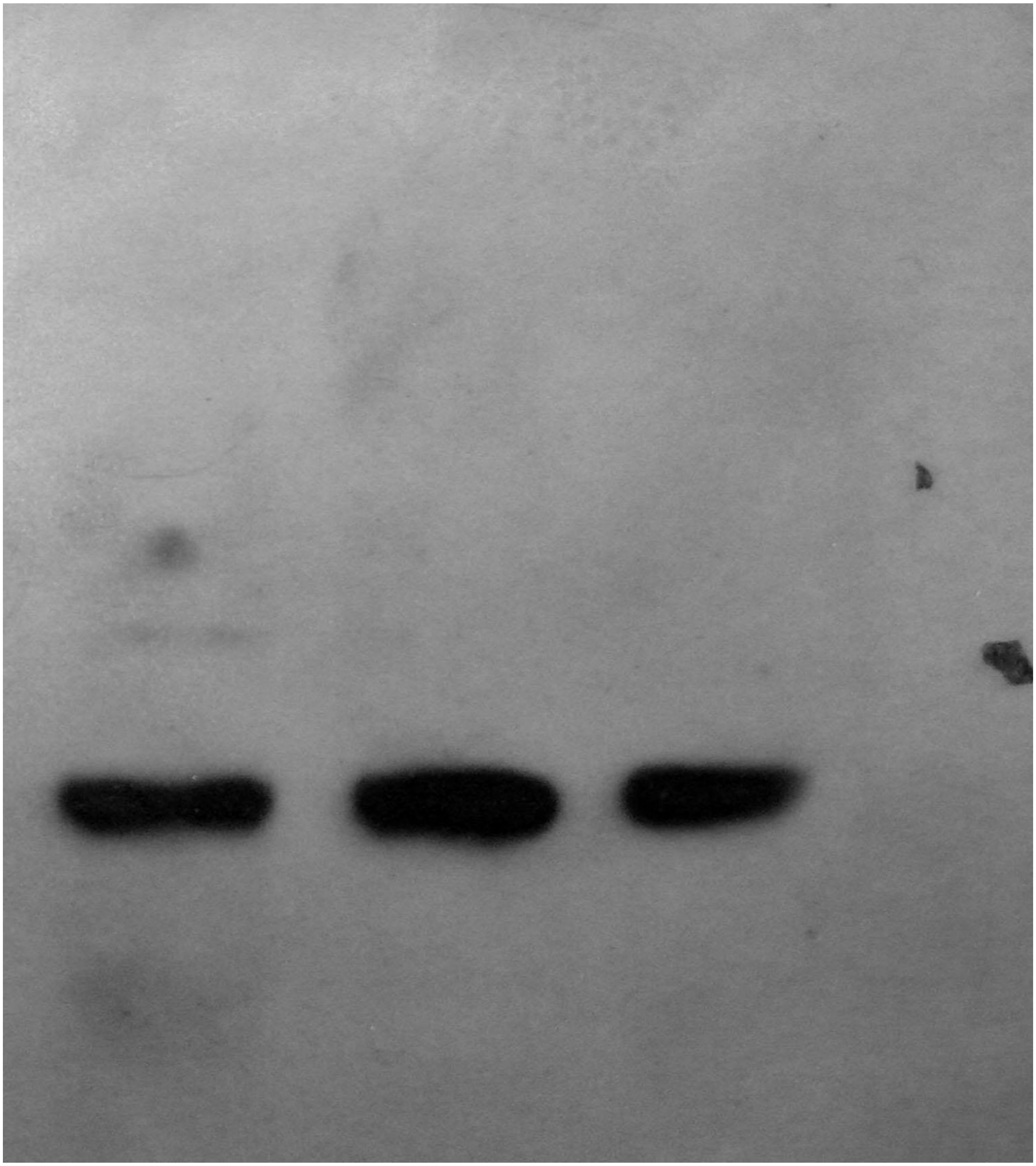

Supplemental Fig. S15: Full length photograph of western blot image with Cleaved caspase 3 expression of isolated hepatocytes.

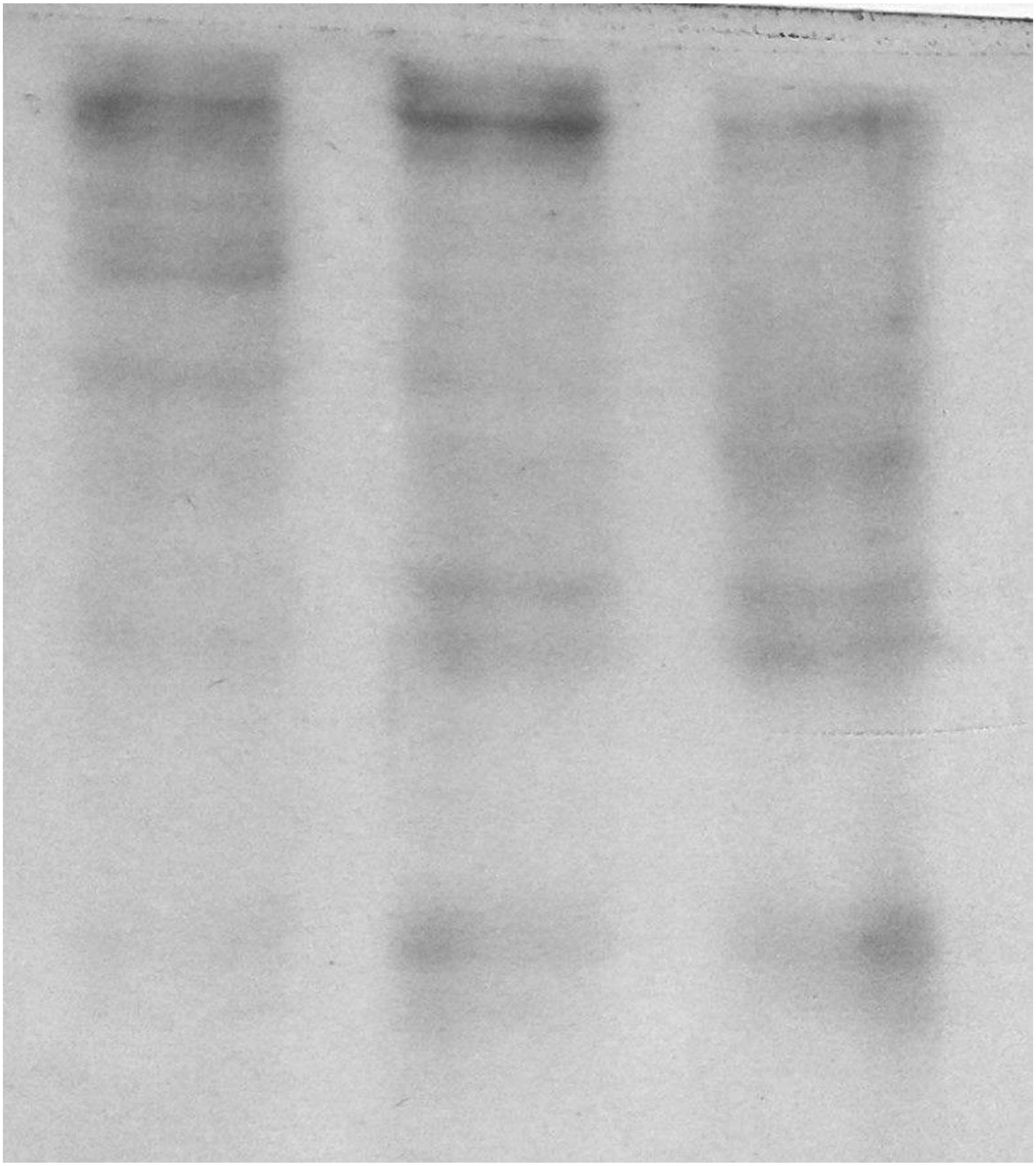

Supplemental Fig. S16: Full length photograph of western blot image with Bax expression of isolated hepatocytes.

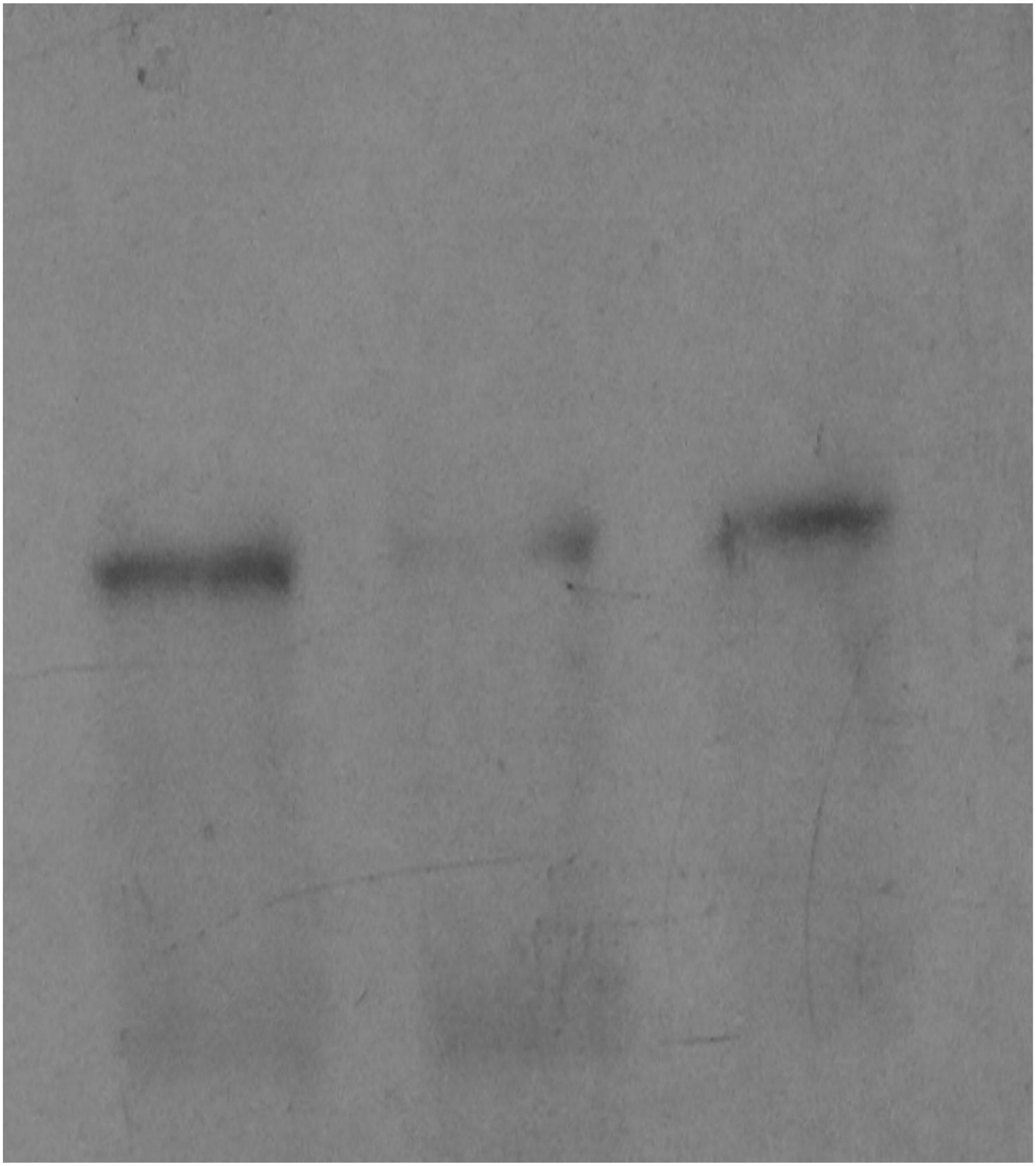

Supplemental Fig. S17: Full length photograph of western blot image with PPAR-α expression of isolated hepatocytes.

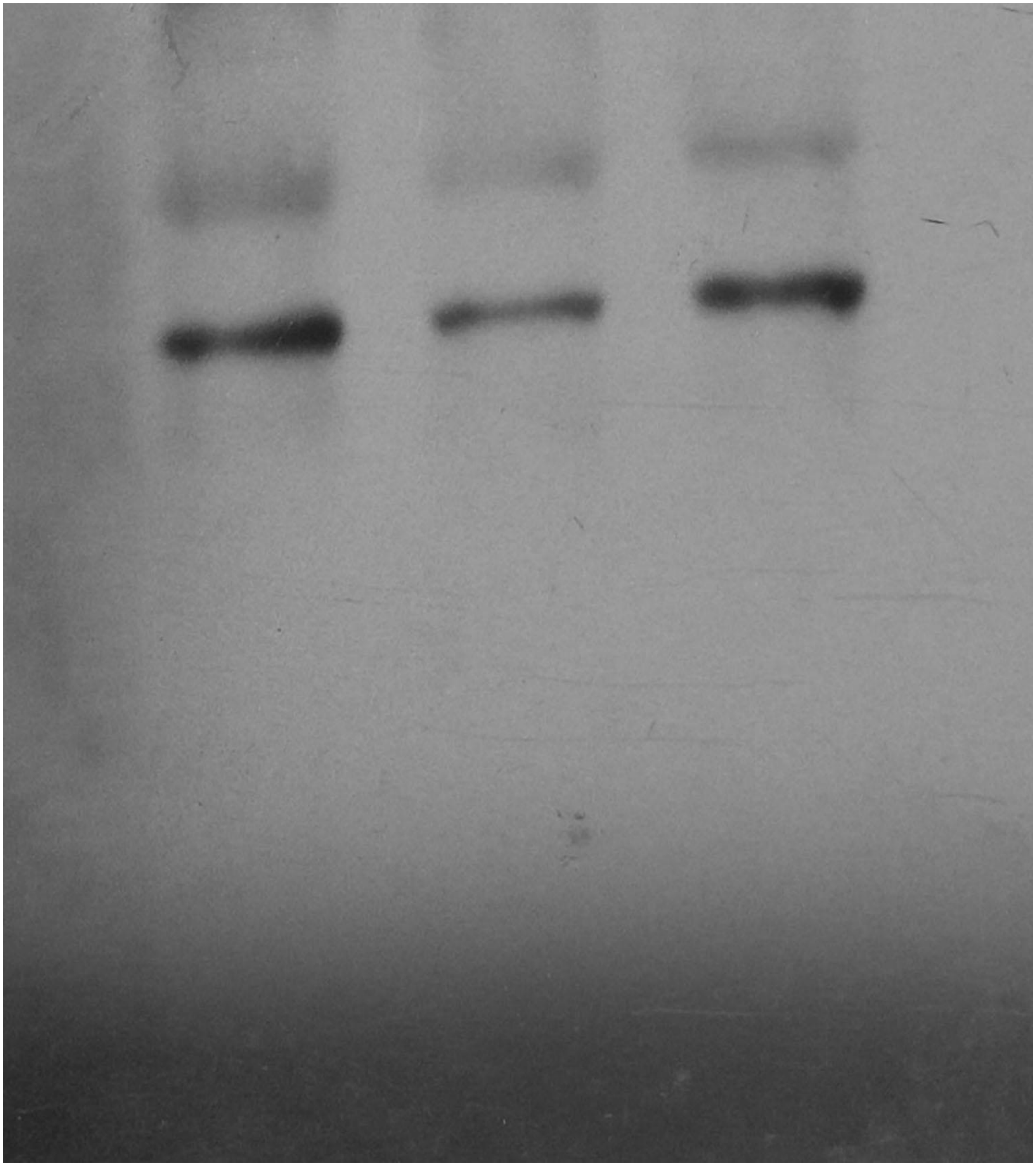

Supplemental Fig. S18: Full length photograph of western blot image with PPAR-γ expression of isolated hepatocytes.

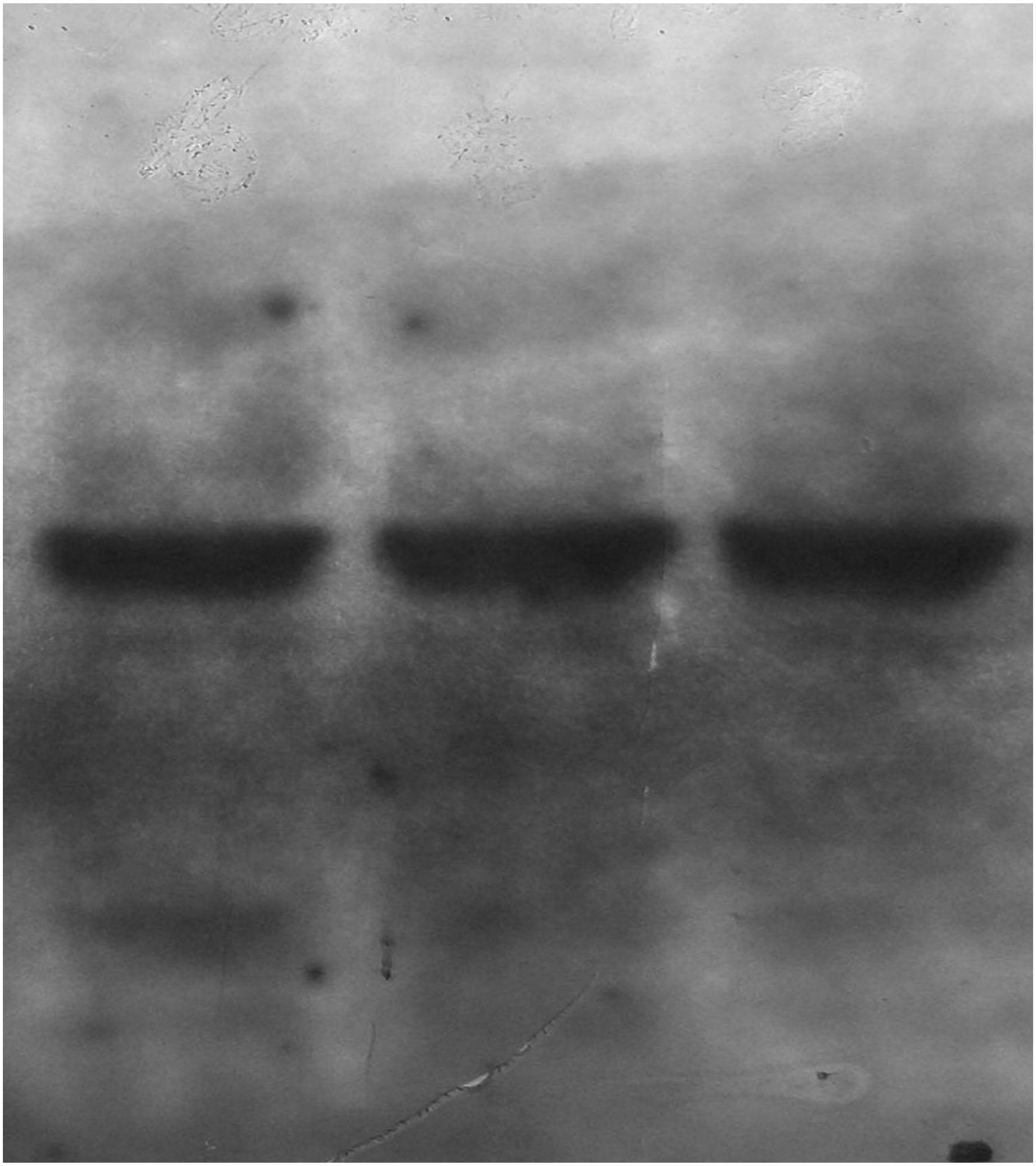

Supplemental Fig. S19: Full length photograph of western blot image with GAPDH expression of isolated hepatocytes.

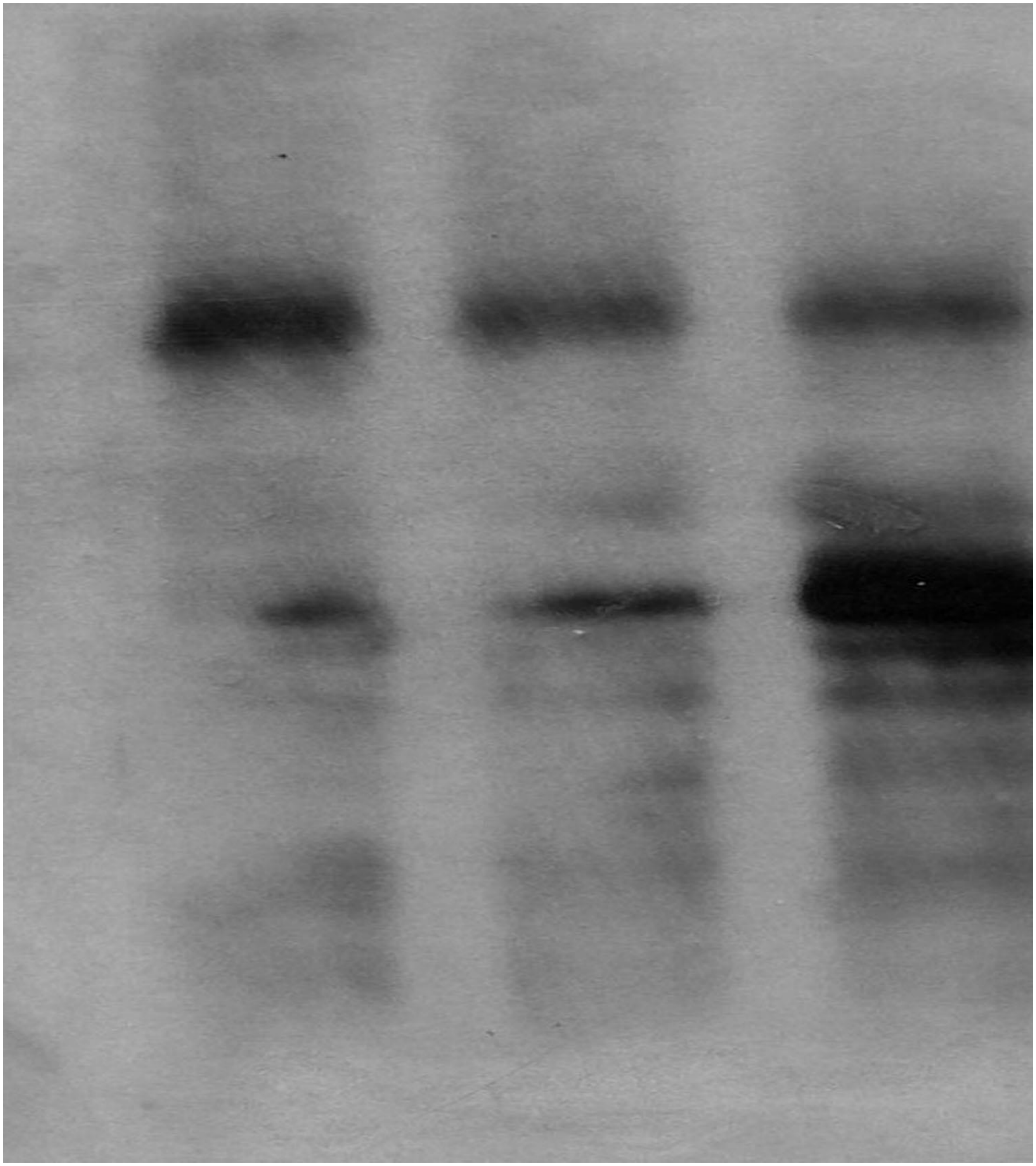

Supplemental Fig. S20: Full length photograph of western blot image with Bcl2 expression of isolated cardiomyocytes.

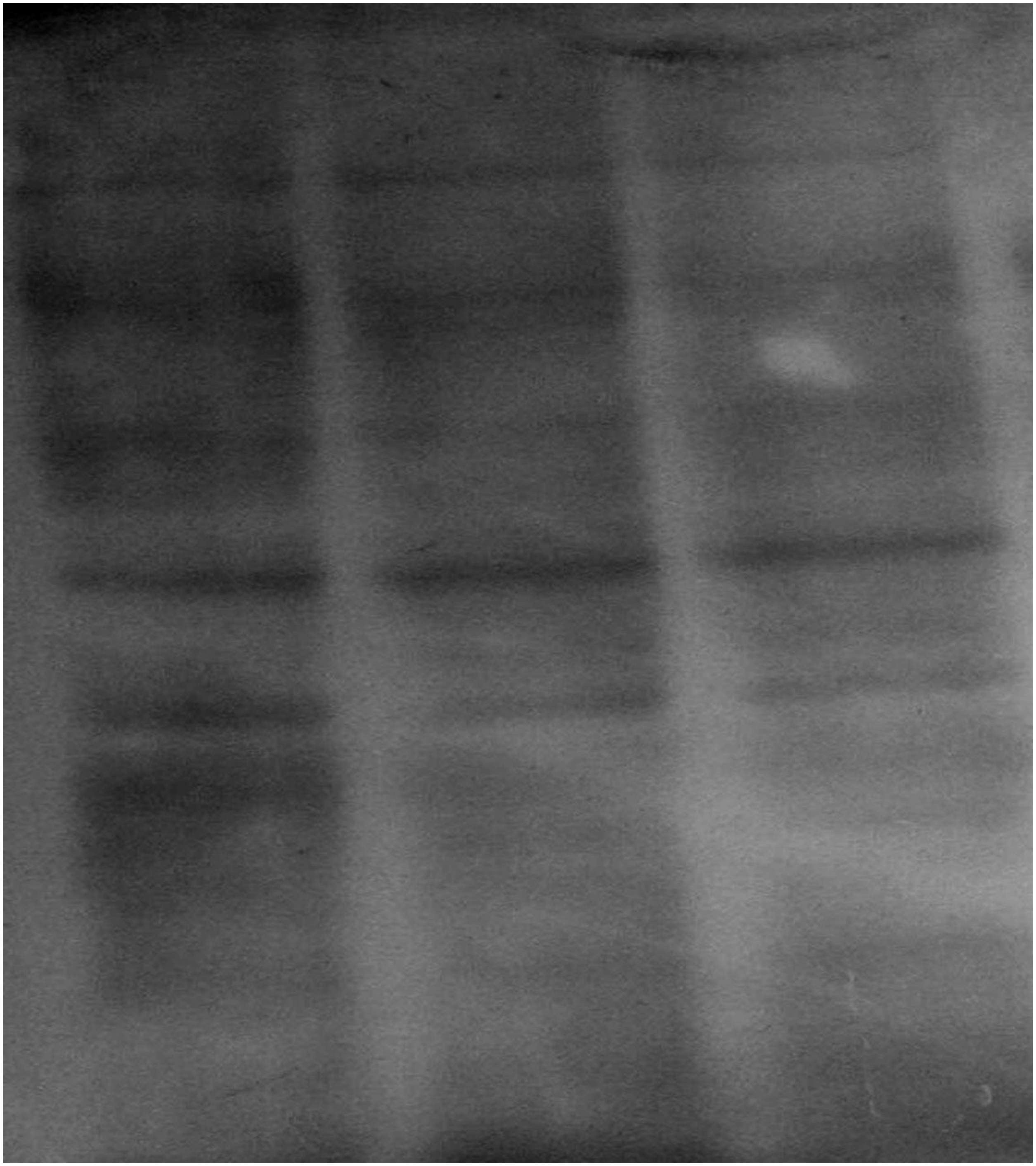

Supplemental Fig. S21: Full length photograph of western blot image with p53 expression of isolated cardiomyocytes.

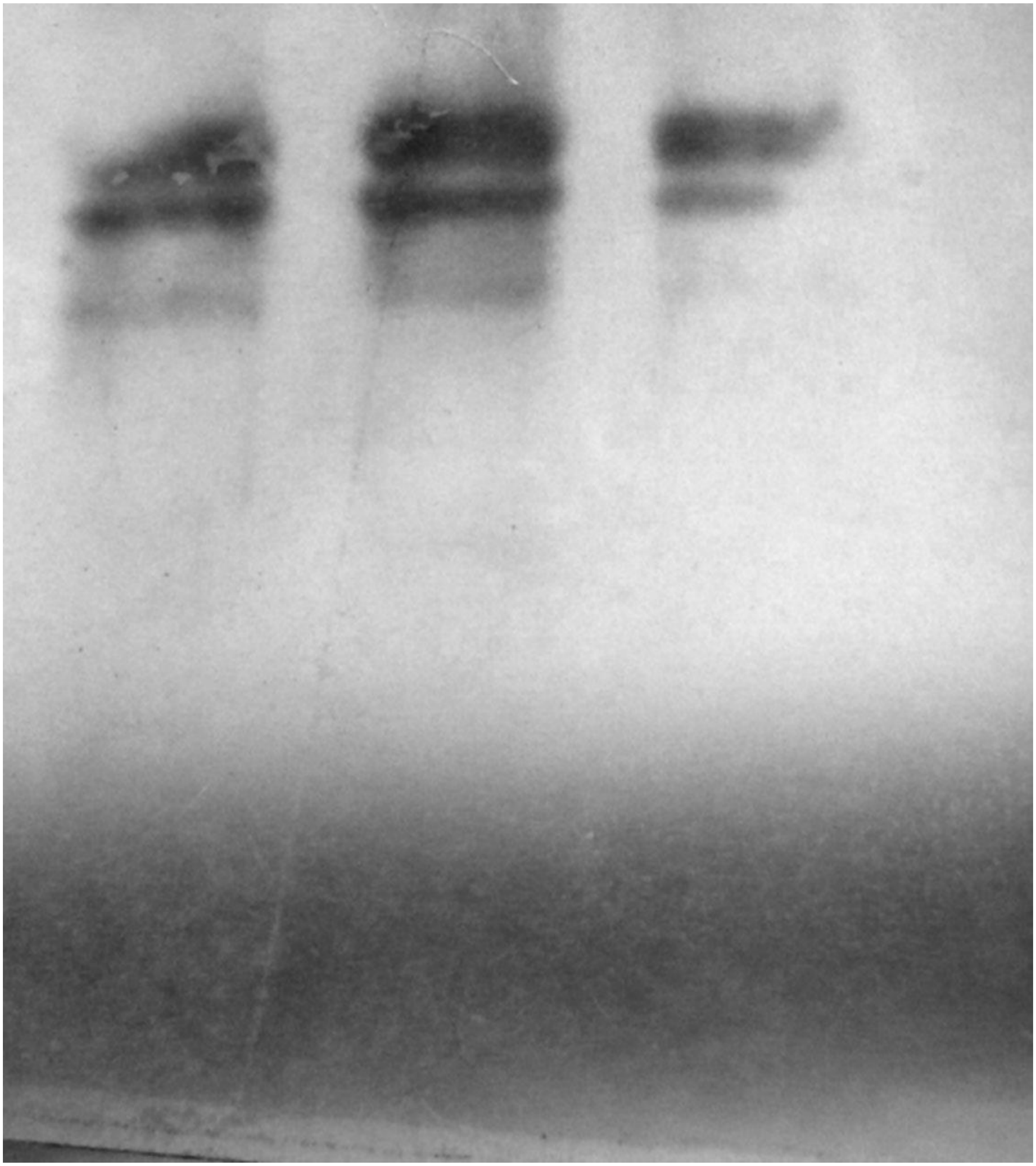

Supplemental Fig. S22: Full length photograph of western blot image with Cleaved caspase 9 expression of isolated cardiomyocytes.

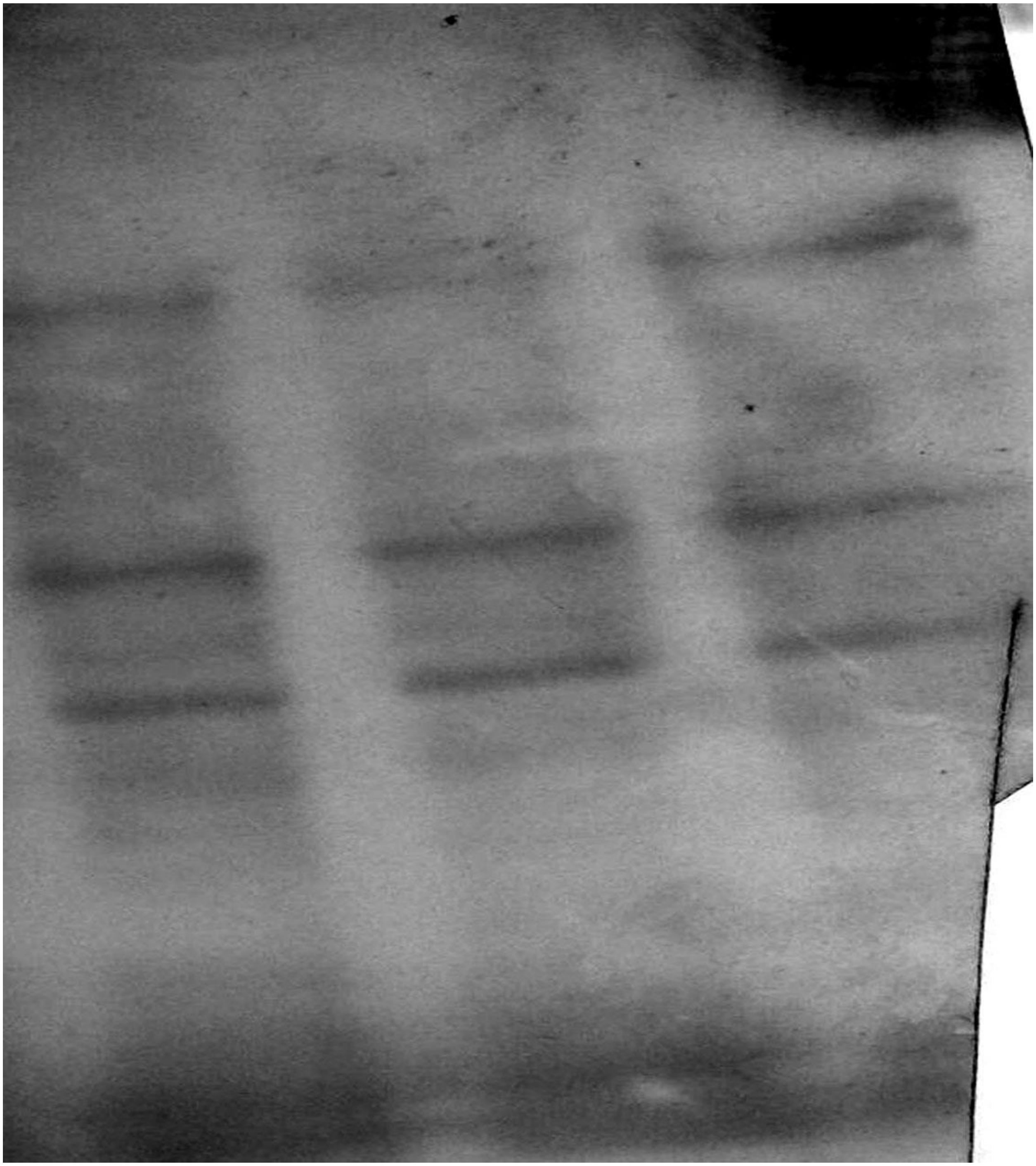

Supplemental Fig. S23: Full length photograph of western blot image with p21 expression of isolated cardiomyocytes.

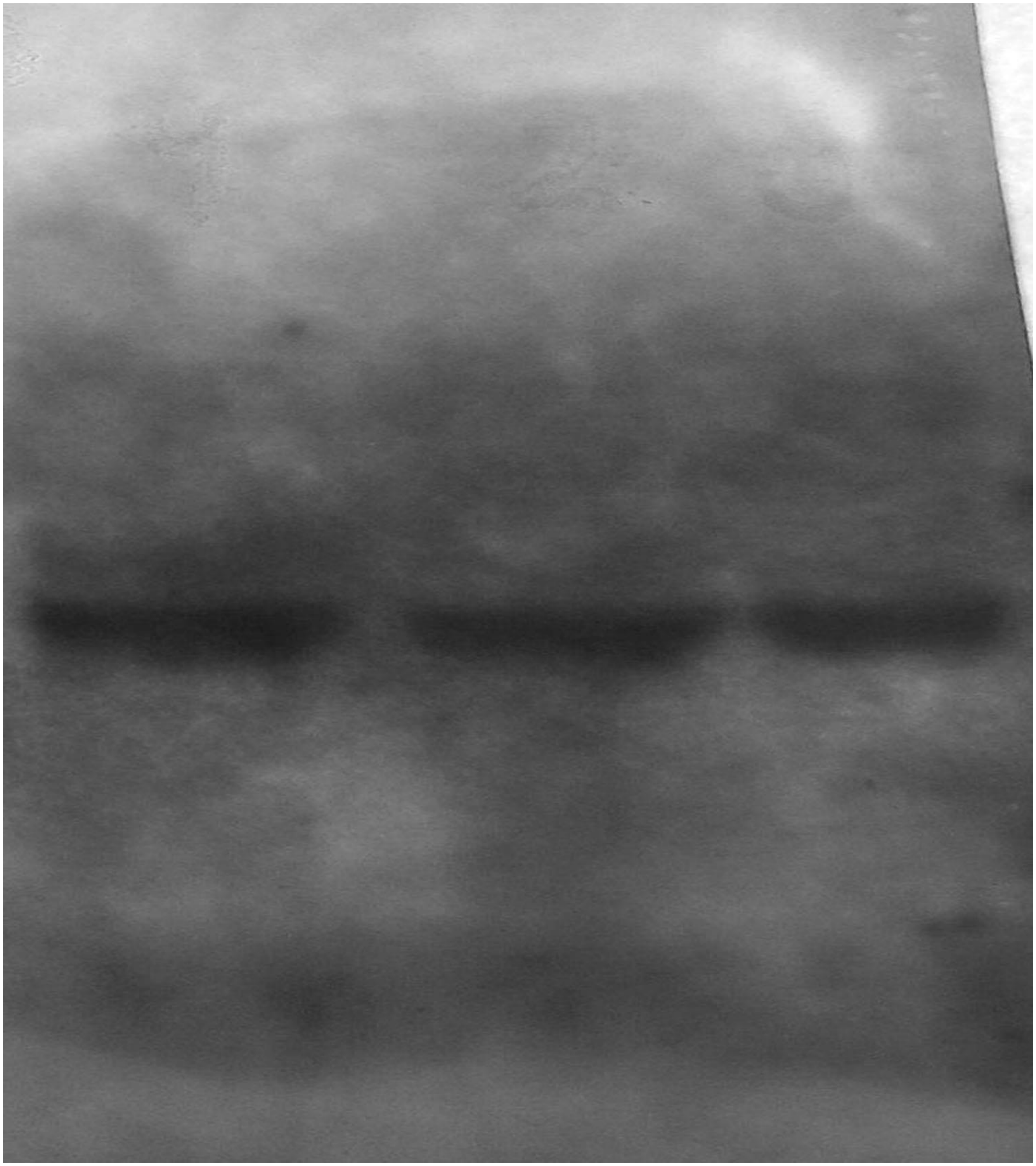

Supplemental Fig. S24: Full length photograph of western blot image with Cleaved caspase 3 expression of isolated cardiomyocytes.

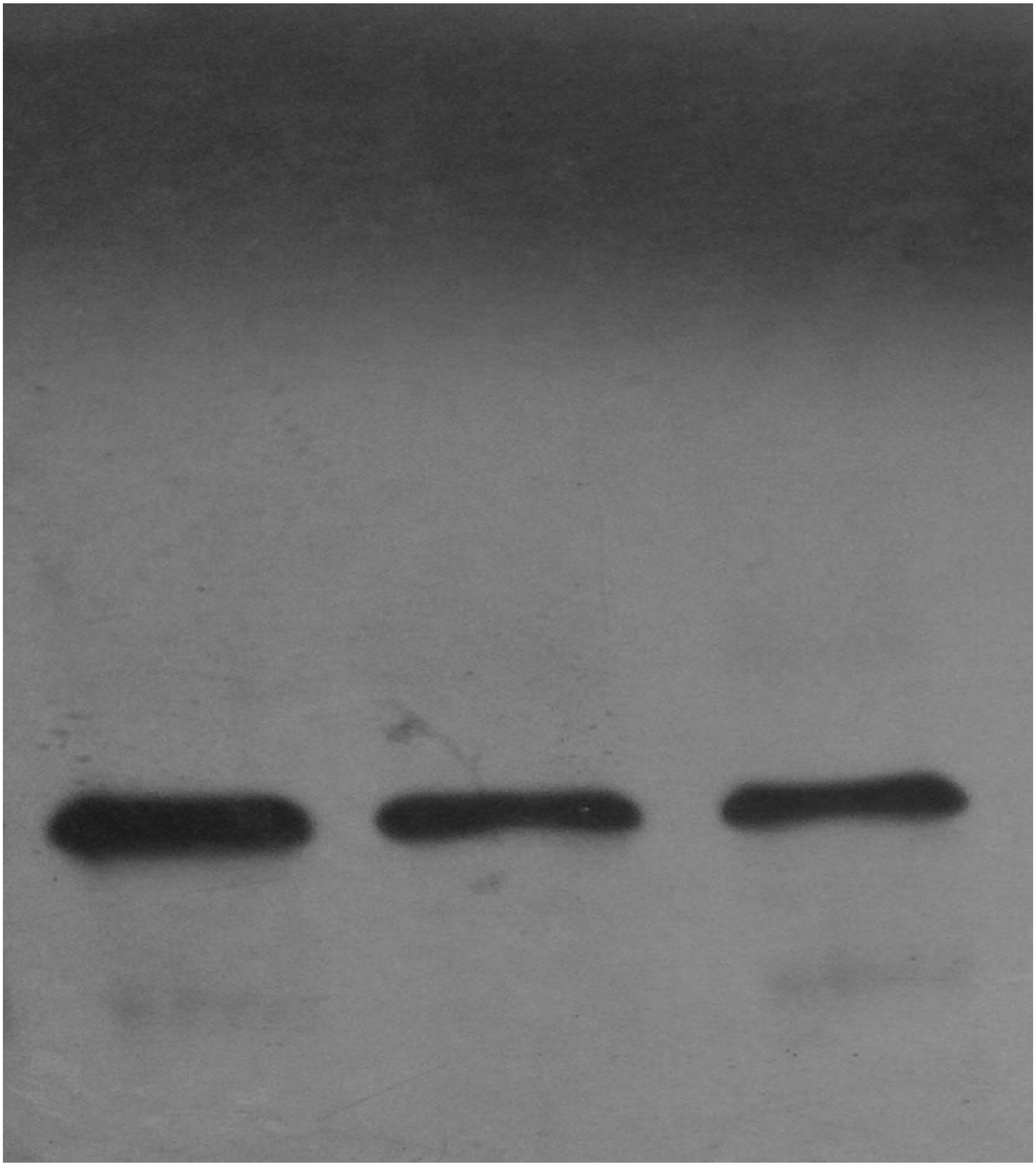

Supplemental Fig. S25: Full length photograph of western blot image with Bax expression of isolated cardiomyocytes.

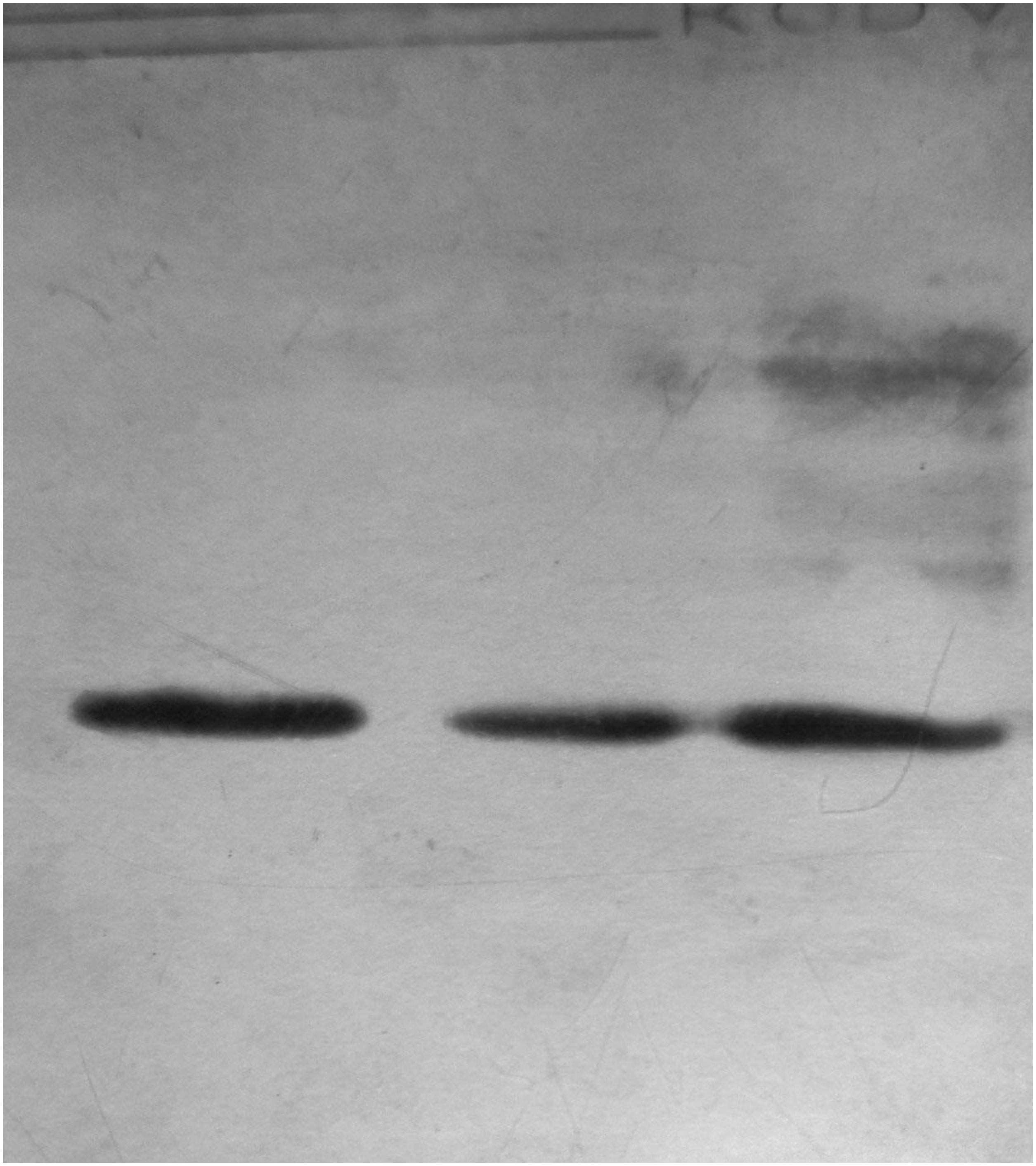

Supplemental Fig. S26: Full length photograph of western blot image with PPAR-α expression of isolated cardiomyocytes.

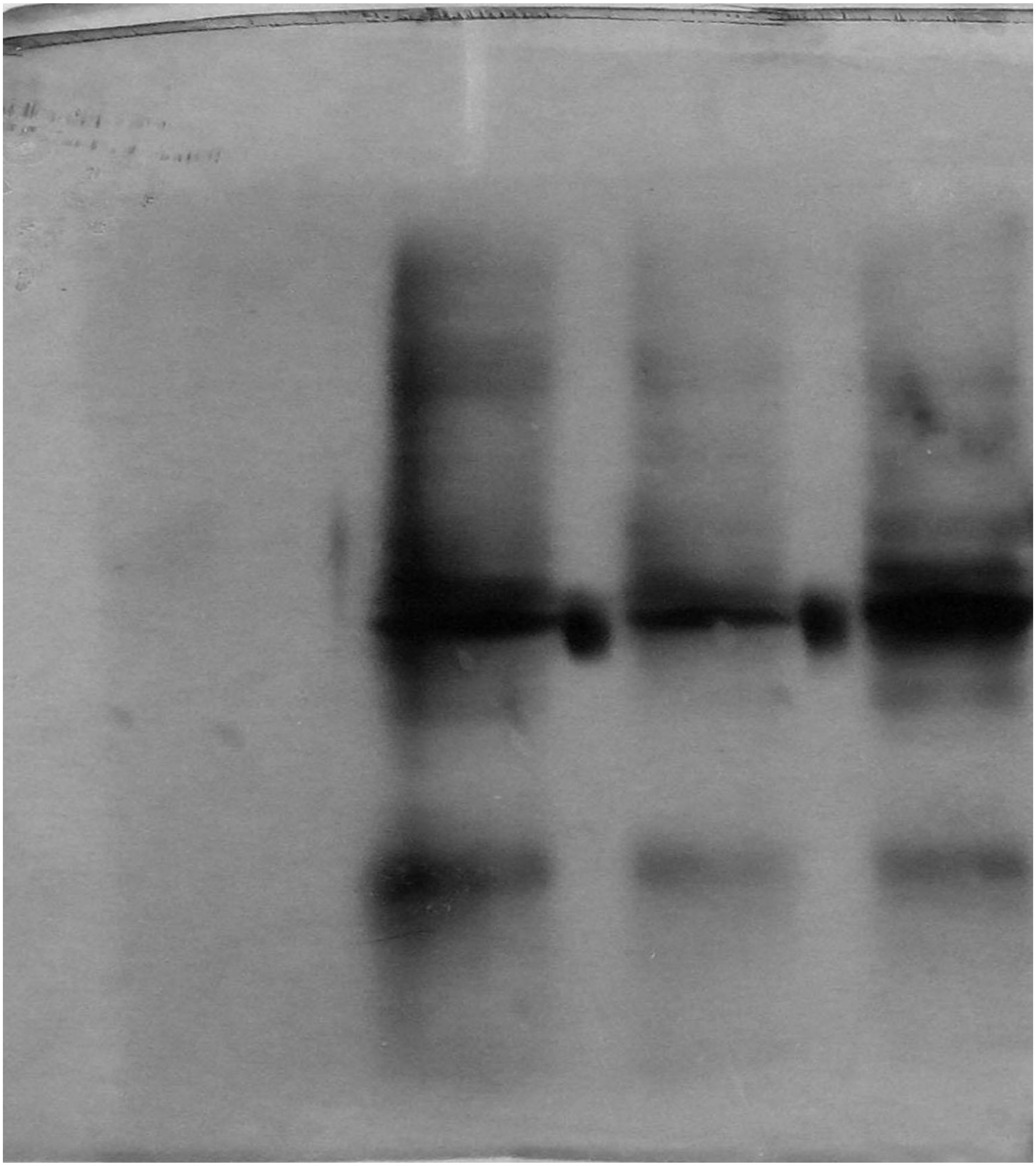

Supplemental Fig. S27: Full length photograph of western blot image with PPAR-γ expression of isolated cardiomyocytes.

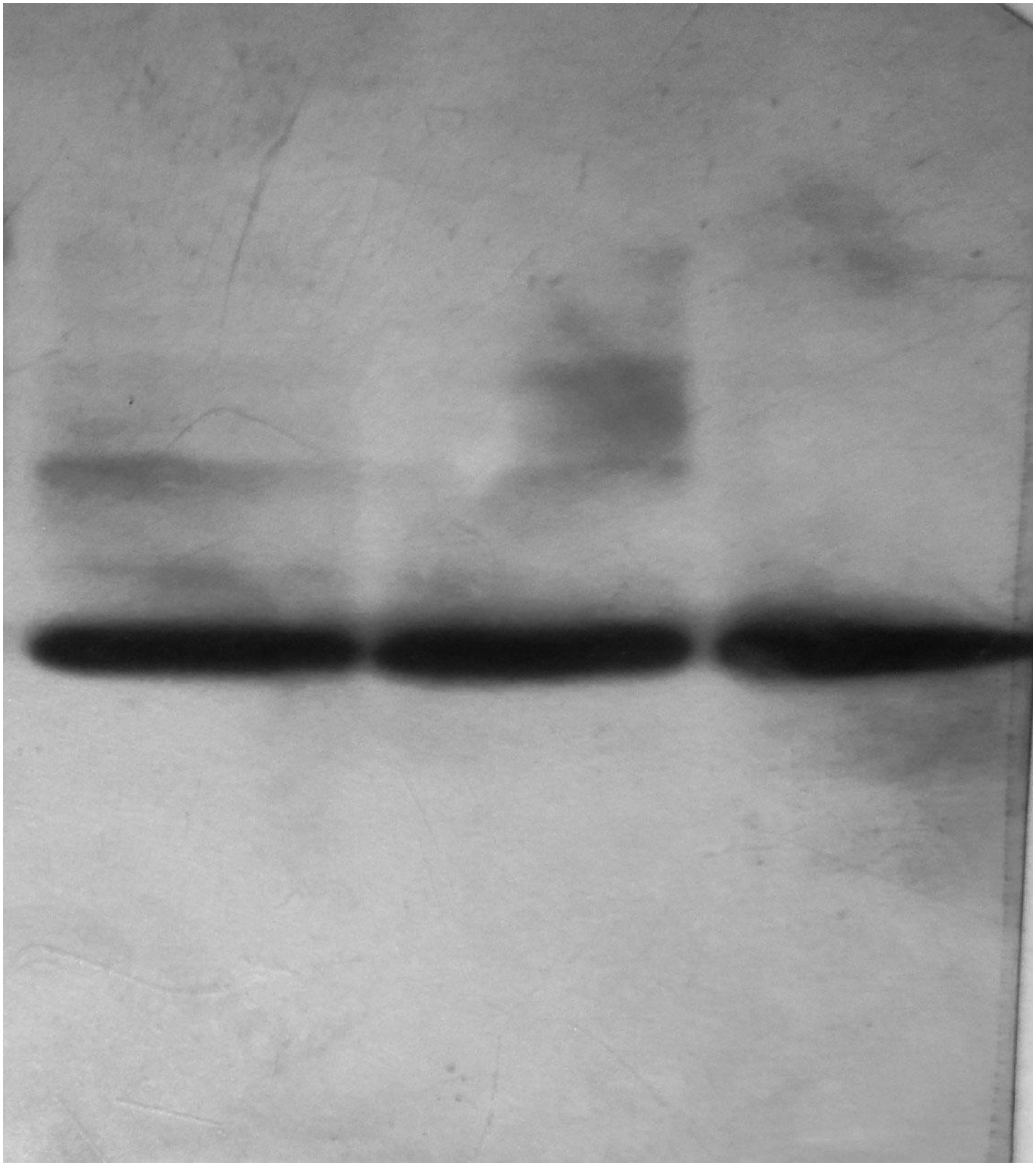

Supplemental Fig. S28: Full length photograph of western blot image with GAPDH expression of isolated cardiomyocytes.
